# Structure and Optical Bandgap Relationship of π-Conjugated Systems

**DOI:** 10.1371/journal.pone.0086370

**Published:** 2014-01-31

**Authors:** André Leitão Botelho, Yongwoo Shin, Jiakai Liu, Xi Lin

**Affiliations:** Department of Mechanical Engineering and Division of Materials Science and Engineering, Boston University, Boston, Massachussetts, United States of America; Jacobs University Bremen, Germany

## Abstract

In bulk heterojunction photovoltaic systems both the open-circuit voltage as well as the short-circuit current, and hence the power conversion efficiency, are dependent on the optical bandgap of the electron-donor material. While first-principles methods are computationally intensive, simpler model Hamiltonian approaches typically suffer from one or more flaws: inability to optimize the geometries for their own input; absence of general, transferable parameters; and poor performance for non-planar systems. We introduce a set of new and revised parameters for the adapted Su-Schrieffer-Heeger (aSSH) Hamiltonian, which is capable of optimizing geometries, along with rules for applying them to any 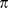-conjugated system containing C, N, O, or S, including non-planar systems. The predicted optical bandgaps show excellent agreement to UV-vis spectroscopy data points from literature, with a coefficient of determination 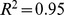, a mean error of −0.05 eV, and a mean absolute deviation of 0.16 eV. We use the model to gain insights from PEDOT, fused thiophene polymers, poly-isothianaphthene, copolymers, and pentacene as sources of design rules in the search for low bandgap materials. Using the model as an in-silico design tool, a copolymer of benzodithiophenes along with a small-molecule derivative of pentacene are proposed as optimal donor materials for organic photovoltaics.

## Introduction

One principal goal of condensed matter theory is to understand the structure-property relationship of materials systems such that specific materials properties may be achieved via molecular design. The emergence of fascinating applications using 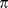-conjugated polymers in light-emitting devices [1] and photovoltaics [2] requires precise control and flexible tuning of optical bandgaps to effectively cover the visible and other parts of the solar spectrum. In particular for photovoltaics, Scharber et al. [3] has derived an empirical formula for the power conversion efficiency (PCE) as a function of the optical gap and the relative LUMO level of the donor material. Existing 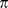-conjugated systems that have optimal bandgaps are still rare. [4], [5].

The optical bandgap of organic molecules can be qualitatively derived from its dependence on the bond length alternation pattern, planarity, aromaticity, and/or electron-withdrawing/releasing substitutions. [4] Although useful in describing certain trends, these properties are ultimately dependent on the electronic structure of the molecule, which must hence be incorporated in any accurate quantitative description. Predicting the optical bandgaps of optoelectroactive materials accurately is challenging because in principle it requires knowledge of the two-particle Green's function propagator. [6] As one of the most successful quantum many-body theories, the first-principles density functional theory (DFT) guarantees only the ground-state properties, [7], [8] and due to the convex nature of the energy functionals at fractional charge states [9] DFT fails to predict fundamental electron localizations in a few strongly correlated materials systems. [10] One of these difficult cases concerns the self-localized soliton and polaron states in conjugated polymers, [11] for which even the time-dependent DFT [12] under the adiabatic local density approximation is known to fail in the extended polymer limit. [13].

Simpler quantum mechanical approaches that are computationally less expensive and can give good bandgap values for specific systems can be divided into two groups. The first group, including for example the Hückel, [14] extended-Hückel, [15] and Valence Effective Hamiltonian, [16], [17] do not properly model the electron-phonon coupling that gives rise to the fundamental excitations (solitons, polarons, and bipolarons) of conducting polymers. Furthermore, these models typically rely on external input from semi-empirical or *ab-initio* calculations for the molecular geometries, [18] defeating the computational cost benefit of using a simpler model.

The second group of models includes electron-phonon interactions and can predict the fundamental excitations of conjugated systems, while simultaneously optimizing the molecular geometries. This group includes the Su-Schrieffer-Heeger model [19] and the related Longuet-Higgins Salem (LHS) model. [20] Although there has been some effort in parameterizing the LHS model for heteroatoms, [21], [22] we are not aware of any study that has validated the transferability of the parameters across more than a few closely related systems.

In our previous works [23], [24], we developed the adapted Su-Schrieffer-Heeger (aSSH) Hamiltonian and demonstrated its accuracy to be better than that of TDDFT, while still correctly describing polarons and their effects, as well as photoinduced charge transfer when 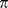-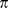 stacking inter-chain terms are added. [25] We have chosen to neglect explicit correlations, which may be needed to describe a few phenomena such as negative spin-density waves [26], multiple gap states [27], and 3D conformation couplings [28], in favor of a simpler model that can be solved quickly through a single diagonalization of the Hamiltonian. The underlying physics is to universally re-normalize the strong electron correlations into the effective electron-phonon couplings so that the phonon-dressed quasi-particles can be created across all these different types of 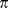-conjugated systems under the Fermi liquid theory. [11], [19].

Nevertheless, until now we had only demonstrated the transferability of the aSSH model for different chain lengths of the very polymers used to determine the parameters. For example, the parameters for sulfur were fit using the optical gap from 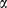-sexithiophene and high level electronic structure calculations for the geometry of the thiophene monomer, but transferability was only shown for the series of oligothiophenes of differing chain lengths. In this work, we not only determine new sets of parameters to extend the scope of the model, but we also demonstrate their transferability by predicting the optical bandgaps for 198 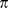-conjugated systems representing a wide range of 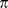-conjugated structures.

To exemplify our intended utility of the model as an in-silico design tool, we focus on the problem of finding an appropriate donor material for bulk heterojunction (BHJ) photovoltaic devices. In these BHJ devices, the electron donor absorbs photons to create electron-hole pairs while the acceptor material draws the electrons but not the holes from the donor. The number of photons absorbed that generate charge carriers directly contributes to the short circuit current (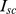) of the device. Since only photons with energy higher than the optical gap can be absorbed, a smaller gap leads to a higher current. However, higher energy excitations eventually relax down to the LUMO of the acceptor, so the open circuit voltage (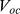) can never be larger than the difference between the LUMO of the acceptor HOMO of the donor. Since the power output, and hence the PCE, is explicitly dependent on 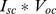, and reducing the optical gap increases 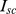 but reduces 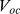, there is an optimal gap for any device that maximizes the PCE. Scharber et al. [3] has derived an empirical formula for the PCE as a function of the donor's optical gap and the relative LUMO levels of the donor and acceptor materials. Assuming a suitably matched acceptor material, the most important intrinsic property to improve PCE becomes the optical gap. From Scharber's analysis, an optimal range for the optical gap lies between 1.2 and 1.5 eV, close to the Shockley-Queisser model result of 1.1 eV for inorganic *p-n* single junction solar cells. [29] Analyzing the aSSH model predictions to understand which structure modifications lead to lower optical gaps, we propose two new 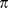-conjugated structures with a potential for the optimal 1.2 eV optical bandgap for single junction devices [3], [29]: one suitable for polymer solar cells [30], [31] and the other for small-molecule crystalline solar cells. [32].

## Computational Methods

The aSSH Hamiltonian [23] used in this work can be written as
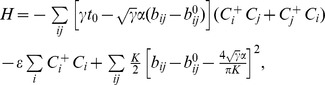
where 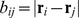 is the bond length between the 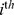 and 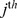 atomic sites located at 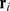 and 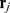 respectively, with the corresponding dummy reference 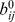. [23] The original SSH constants include the 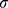-bond spring constant 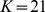 eV/Å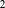, the reference hopping integral 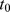 = 2.5 eV, and the linear electron-phonon coupling constant 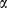 = 4.1 eV/Å. [19] 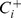 and 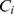 are the creation and annihilation operators for the 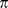-electron at site 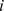, respectively. The dimensionless 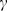 scales the hopping integral parameters as 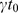 and 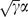 so that 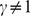 for all bonded pairs that include heteroatoms or are part of six-membered aromatic rings. Such a choice of electron-phonon scaling ensures that the universal electron-phonon coupling constant [11] 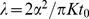 and the 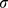-bond spring constant 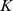 are unaltered for all conjugated bonds, which means all of the 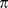-electrons are treated on an equal footing. 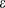 denotes the density operator 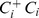 strength so that 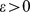 attracts more electrons onto the heteroatomic sites as compared to the carbon reference where 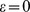.

The fully transferable 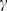 and 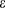 values summarized in Table 1 are used for all the 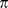-conjugated systems discussed in this work. Except for the polymer-fullerene heterojunctions, our calculations are performed on finite-sized, isolated molecules and compared with the optical bandgaps from oligomers and polymers in dilute solutions (with only PBDTDTBT [33], [34] and PFDTTPDB [35] from thin film), for which the inter-chain steric effects are minimized. The heterojunctions require additional 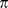-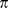 stacking Hamiltonians as discussed separately by Shin and Lin. [25] The determination of the parameters for 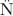, 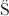, and benzene bonds is discussed in our previous works [23], [24], while the values for Ö we now re-determine using the widely overlooked lowest energy UV-vis absorption peak from polyfuran (PFu). [36] Newly determined for this work are the parameters for 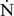 using pyridine, [37] 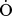 using thieno[3,4-c]pyrrole-4,6-dione polymer, [38] and the bridge 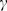 for perpendicular fused rings using poly-isothianaphthene. [39], [40] The new parameters for heteroatoms are unambiguously determined using the same methodology as our previous work, namely using a single optical gap point from a well characterized oligomer and the bond dimerization magnitude from a monomer calculated with either the quadratic configuration interaction with single and double substitutions (QCISD). [41] The new parameter for bridge bonds, which are dependent on the structure of the connected monomers, is discussed under the perpendicular fused rings section of the results and discussion. We also change the parameters for poly-(*p*-phenylene vinylene) (PPV), [42] poly-(*p*-phenylene) (PPP), [43] and polyacenes[44]–[50] to 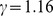 and 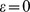 from benzene in order to simply the rules for applying the model, where these parameters are now applied to any 6-membered aromatic carbon ring, regardless of whether it is benzene, a phenyl group, or it is fused with other rings. In the case where a bond is part of two fused rings, if one of the rings is a six-membered carbon ring the parameters from benzene are still applied. The 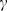 parameter for any bond between two heteroatoms is taken as the 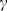 from the heteroatom with the heavier mass.

**Table 1:** aSSH parameters.

	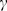	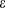 (eV)
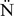	1.47^a^	4.36^a^
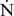	1.05	0.59
Ö	1.44	5.46
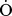	1.07	0.34
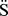	1.15^a^	2.85^a^
Benzene	1.16^a^	0^a^
Bridge between for perpendicular 2-rings	0.75	–
Bridge between perpendicular 3-rings	0.20	–
All other 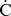	1^b^	0^b^

Dots over elements specify the number of 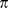-electrons contributed to the conjugated system. C-C bonds in six-membered aromatic rings and the bridge bonds between perpendicular fused rings require 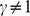.

a Reference: [24].

b Original parameters from [19].

## Results and Discussion

**Figure 1:**
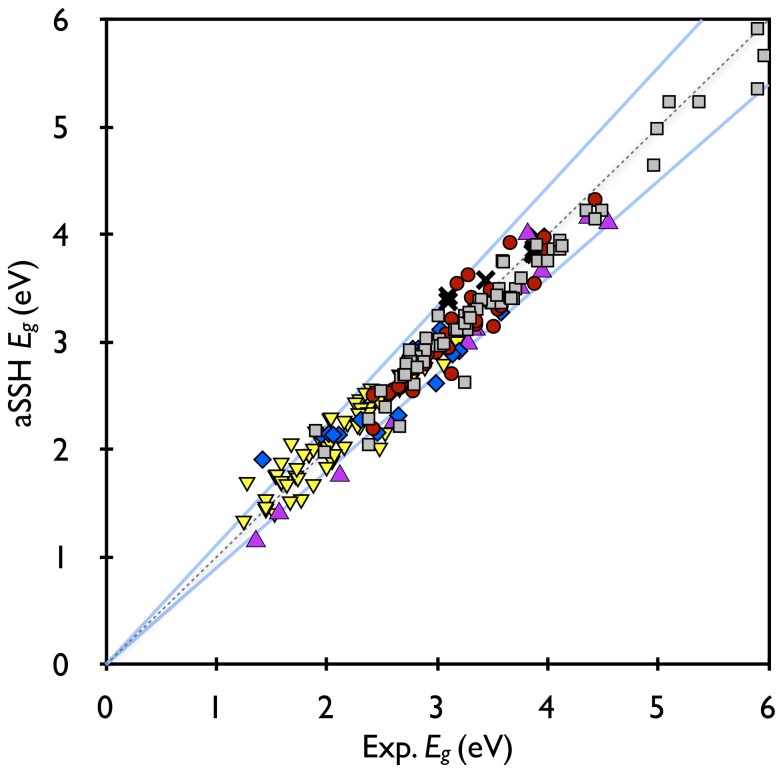
Predicted aSSH optical bandgaps are compared with experimental ones for 198 independent 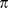-conjugated systems. Subgroups include simple rings (Gray squares), parallel fused rings (red circles), perpendicular fused rings (blue diamonds), copolymers (yellow down-triangles), PAHs (violet up-triangles), and 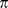-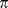 stacking systems (gray crosses). Dashed and solid lines are 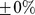 and 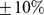 deviations from experimental values, respectively. The coefficient of determination of 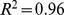, mean error −0.05 eV, and mean absolute deviation 0.16 eV.

**Figure 2:**
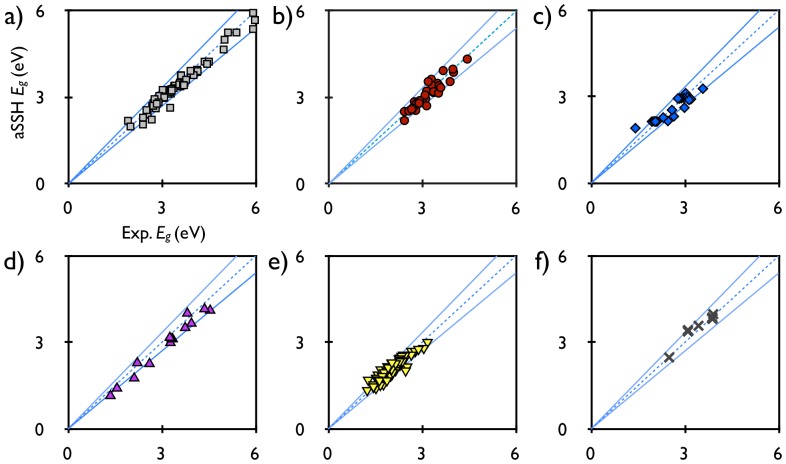
Subgroups of calculated bandgaps compared to experimental: a) simple rings, b) parallel fused rings, c) perpendicular fused rings, d) copolymers, e) PAHs, and f) 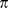-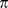 stacking systems. Dashed and solid lines are 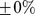 and 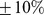 deviations from experimental values, respectively.

Since all of the experimental data are taken from UV-vis absorption spectra reported in literatures, we will focus solely on the optical bandgaps as opposed to either the electronic or transport bandgaps. Fig. 1 shows the agreement between the optical bandgaps predicted by the aSSH model and those measured by UV-vis experiment, where perfect agreement lying on the central dashed line and 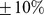 within the solid lines. The predicted bandgaps have an overall coefficient of determination of 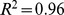, a mean error 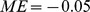 eV with a mean absolute deviation of 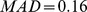 eV. Numerical tables of all the calculated versus experimental optical gaps along with references and chemical structures can be found in the Supporting Information, Tables S1–6 and Figures S1–6. The 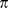-conjugated systems summarized in Fig. 1 are grouped into six categories according to their constituent monomer units, each shown individually in subplots in Fig. 2: a) simple aromatic rings (gray squares) such as polythiophene (PTh), polypyrrole (PPy), PFu, PPP, PPV, and *etc.*; b) fused aromatic rings parallel to the conjugated path direction (red circles); c) fused aromatic rings perpendicular to the conjugated path direction (blue diamonds); d) copolymers from polymerization of any of the previous groups (yellow triangles pointing downwards); e) polycyclic aromatic hydrocarbons (PAHs) including polyacenes where the conjugated paths are greater than one dimension (violet triangles pointing upwards); and f) systems with explicit interchain 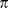-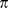 interactions with fullerene and PPV bulk structures (gray crosses). Guided by insights from each category, we make recommendations for both small molecule and polymer optoelectro-devices operating at the 1.20 eV bandgap target, [3], [29] as well as 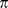-conjugated materials with bandgaps as low as 0.8 eV for long wavelength absorbing materials used in tandem cells.

### Simple Aromatic Rings

The agreement between the calculated and experimental gaps are shown in Fig. 2a and has the best fit of any of the groups here, with 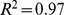, 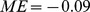 eV, and 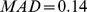 eV. Out of the 67 included here, one point for each of PTh, PFu, PPy, and pyridine were used as targets for fitting 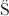,Ö,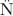, and 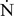, respectively, and hence have zero error. Removing these points from the data set increases the 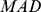 by 0.01 eV but has no significant effect on 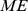 or 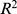. Considering the extended polymer limit, for which the 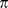-conjugation length 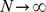, the optical bandgaps decrease from 3.64 eV for PPP, 3.05 eV for PPy, 2.83 eV for PFu, 2.63 eV for PTh, to 2.54 eV for PPV. [24] Further decreases in the bandgaps have been pursued through structural modifications, [4] typically starting from PTh and PPV as they have the lowest bandgaps of the group, in an effort to achieve the optimal gap of 1.20 eV. [51].

**Figure 3:**
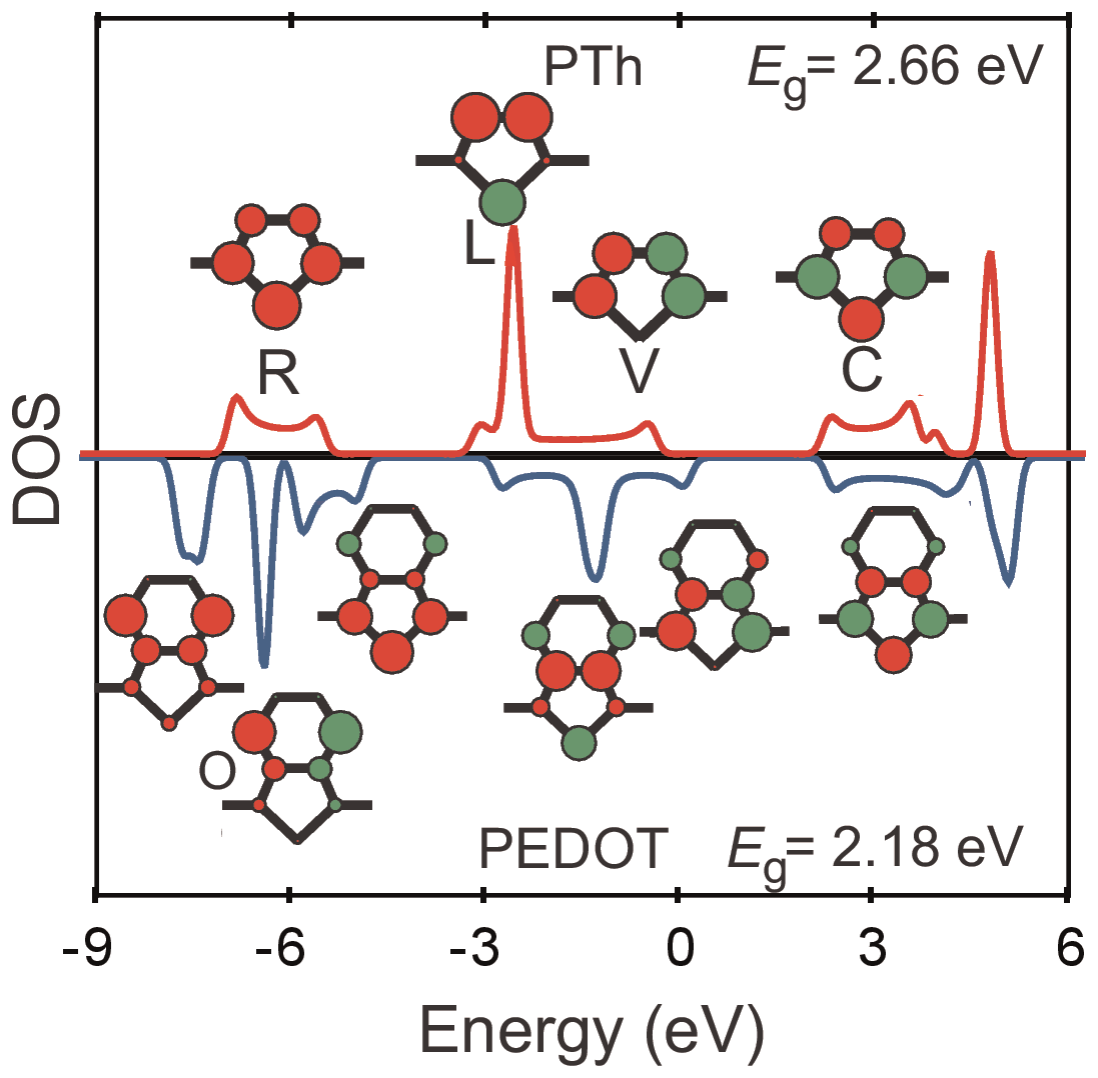
Density of states (DOS) and wavefunctions of all the 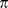-bands of PTh and PEDOT, both containing 20 monomer units. In addition to the ring (R), localized (L), valence (V), conduction (C), and other bands of higher energies (wavefunctions not shown) of PTh, the two low-lying oxygen (O) bands that are formed in PEDOT push the remaining bands, which have nodes between the O and 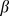-C sites, upwards in energy. The HOMO level (top of the V band) increases in energy more than the LUMO level (bottom of the C band) because the former has larger wavefunction components on the 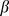-C sites. This effectively lowers the optical bandgap of PEDOT, as compared to PTh.

One straightforward way to modify the bandgap of PTh is to replace the 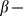
hydrogens with side-groups that will affect the 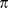-conjugation. Such is the case of the widely used poly-3,4-ethylenedioxythiophene (PEDOT), in which two oxygens are conjugated to the two 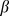-C sites of PTh. Note that PEDOT is different from the perpendicular fused aromatic rings (to be discussed below) in that these oxygens are not part of an aromatic ring. As shown in Fig. 3, the lower optical bandgap of PEDOT, 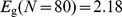 eV as compared to 2.66 eV for PTh, is caused by the two new oxygen (O) bands lying below the ring (R) band [23]. These two O bands consist of all the 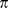-binding states between the O and 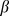-C sites, which push the remaining anti-bonding states, i.e. O and 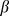-C having opposite phases, upwards in energy. Because the valence (V) band has larger wavefunction components on the 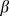-C sites than the conduction (C) band, the corresponding highest occupied molecular orbital (HOMO) of PEDOT moves upwards in energy more than the lowest unoccupied molecular orbital (LUMO). Therefore, the bandgap of PEDOT decreases.

### Parallel Fused Rings

Starting from PTh, one popular way in which researchers have been attempting to lower the bandgaps is to fuse adjacent rings by adding extra conjugated sulfur bridges. [4] The argument commonly used to justify this idea is planarization of the 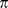-conjugated path, which may be misleading because a system with a non-planar configuration is indicative of weak electron hopping across long bridge bonds [52] and does not necessarily imply a large bandgap.

**Figure 4:**
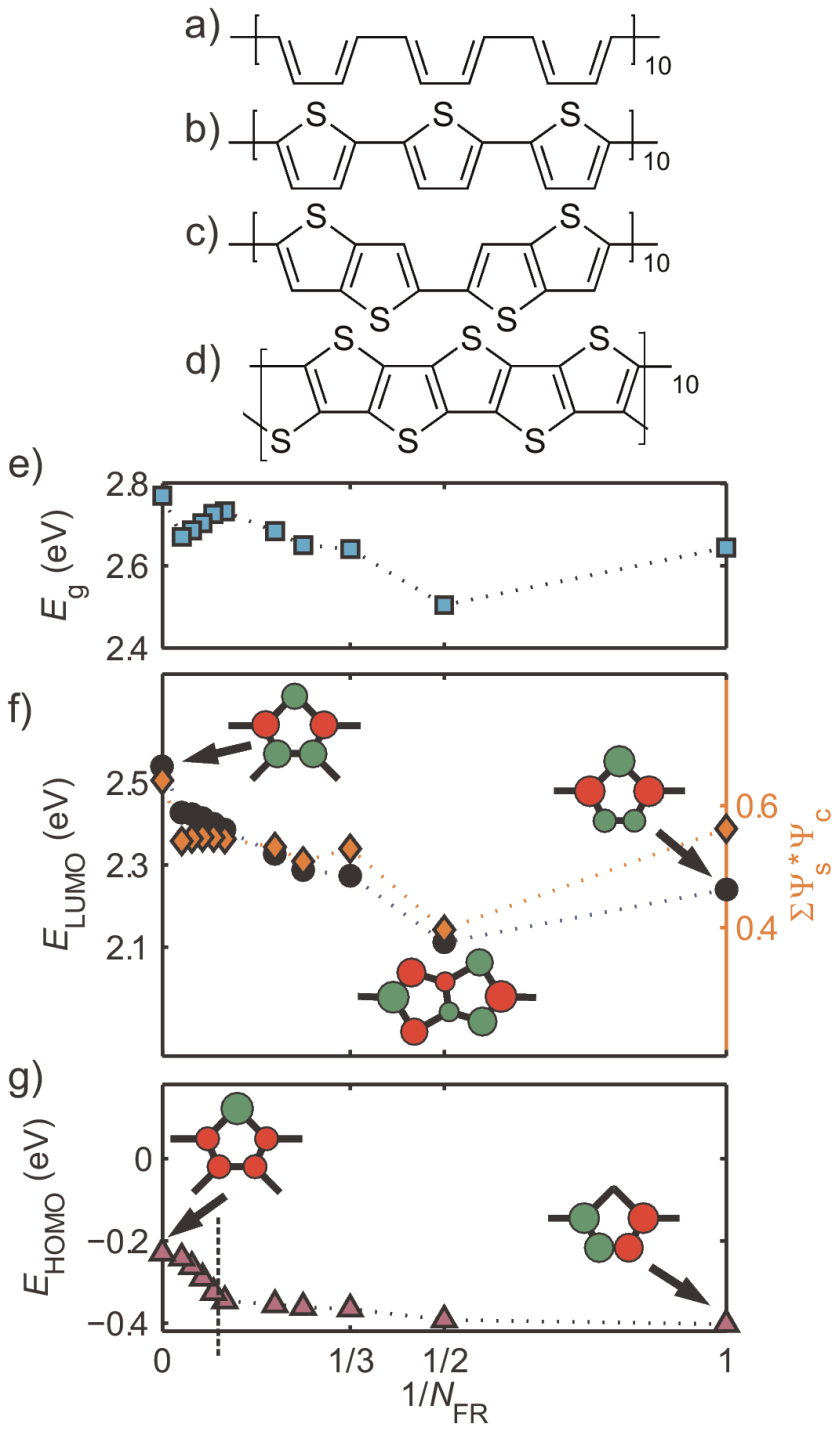
PTh (b), PT32bT (c), and PTA (d) share the identical conjugated carbon backbone (a) with an equal amount of carbon atoms along the conjugated path, for instance 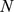 = 120 as shown. The computed optical band gap (e) and energies of LUMO (f) and HOMO (g) as a function of the number of fused rings in their oligomers, where 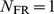 for PTh, 2 for PT32bT, and 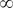 for PTA. Unfavorable electron hopping between S and 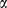-C are plotted as orange diamonds in (f) on the right y-axis.

For a given 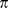-conjugation length, say 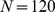 as depicted in Fig. 4a, one may vary the number and positioning of sulfur groups to construct a series of polymers with different numbers of fused thiophene rings 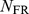 in monomers. For example, PTh of 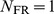 (Fig. 4b) can be seen as poly-thienoacene (PTA) of 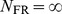 (Fig. 4d) after removal of every other sulfur atom; removing every third sulfur atom in PTA gives poly-thieno[3,2-b]-thiophene (PT32bT) of 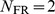 (Fig. 4c); etc. Experimental data from polymers including one, two and three thiophene rings in a single monomer, as well as short PTA's, are compared with aSSH calculations in Fig. 2b and have 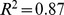, 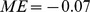 eV, and 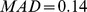 eV.

It is clear from Fig. 4e that the bandgap variation for conjugated polymers consisting of parallel fused thiophene rings does not follow the planarization argument. Instead, we find that PT32bT has the lowest bandgap and PTA has the highest while PTh is essentially in the middle. At this point, one may be tempted to put planarization aside and recall that the bandgap of *trans*-polyacetylene (tPA), the prototypical conducting polymer, is proportional to its bond-length dimerization amplitude. [11] This simple rule, however, does not hold for conjugated polymers containing aromatic rings or even more complicated conjugated structures. [23] The bond-length dimerization amplitude does not follow the same trend and thus cannot satisfactorily describe it. Such non-monotonic variation in 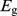 is caused by the wavefunction symmetries and energy band crossovers of the LUMO and HOMO levels as follows.

Since PTh has a LUMO orbital (Fig. 3, C band) with large wavefunction components on the sulfur, which is anti-bonding to the two neighboring 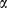-C sites, 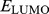 is sensitive to the total amount of such unfavorable electron hopping. As shown in Fig. 4f, PT32bT of 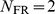 and other polymers of even 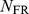 manage to decrease such unfavorable electron hopping through a reduction in the wavefunction components on the 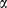-C sites away from bridge bonds. However, 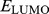 inevitably increases after a couple of odd and even alternations of 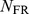. In the 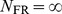 limit, 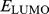 and consequently 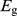 experience a sudden energy jump due to the lack of favorable electron hopping across bridge bonds in PTA.

Opposite to the LUMO case, the HOMO wavefunction of PTh (Fig. 3, V band) has nodes on the S sites. Therefore, increasing 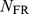 by adding more sulfur sites does not change 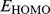 as much as 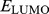. However, as 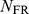 increases, the 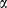-C wavefunction components in the L band (Fig. 3) also increase such that the L band starts to delocalize and its energy becomes dispersed. As the energy dispersion of the L band increases, it eventually overtakes the V band and becomes the new HOMO state. Such an L-V band energy crossover occurs around 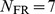 for our 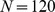 case, after which 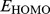 increases sharply (Fig. 4g).

In summary, since the largest 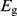 drop is 0.14 eV from PTh to PT32bT (Fig. 4e), the intention to lower the optical bandgap of PTh by using fused rings parallel to the conjugated path would not reach the 1.20 eV bandgap target. As a pleasant surprise, however, the enhanced wavefunction components on the bridge C sites, similar to the PT32bT case shown in Fig. 4f, help greatly in reducing the bandgaps of copolymers as will be discussed in the copolymer section.

### Perpendicular Fused Rings

The predicted optical gaps for this group are shown in Fig. 2c with 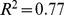, 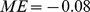 eV, and 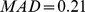 eV. The worst result is for poly-2,3-dihexylthieno[3,4-b]pyrazine (DHTP) [53], with a calculated optical gap nearly 35% higher than from experiments. The poor result of DHTP comes from a combination of two factors: 1) the experiments were done with long polymers that have a tendency to aggregate and thus introduce inter-chain effects and 2) despite attempts to chemically dedope the polymer, it remained paramagnetic, indicating a residual doping that decreases the measured gap.

The important contribution of the perpendicular fused rings series to the aSSH model is in determining a new bridge bond 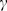 that is dependent on the structure of the monomers being connected. To understand the need for this, we can take poly-isothianaphthene (PITN) as an example. The lower optical bandgap of PITN as compared to PTh is typically attributed to the quinoid (reversed bond orders as compared to aromatic) character of the PITN backbone. [54] This extends from a comparison to tPA systems in which an “aromatic” and a “quinoid” dimerization (which in tPA are degenerate) are connected in reaction space by a non-dimerized form with a vanishing gap. Following this idea, simply reducing the aromatic or quinoid character towards a non-dimerized backbone would lead to a reduction in the gap. It follows that if the dimerization of the backbone is responsible for lowering the gap, we should expect the gap to lower at a faster rate with increasing chain (backbone) length. This trend is not what we observe between PTh and PITN.

**Figure 5:**
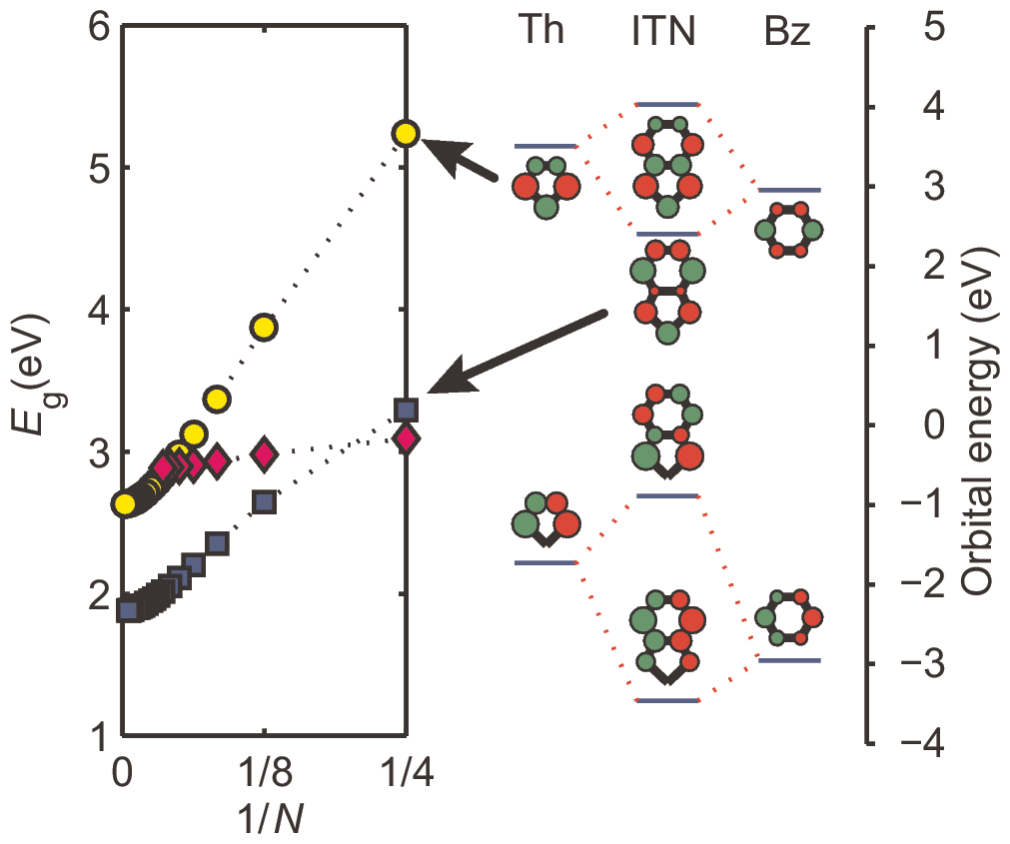
Bandgap as a function of the 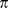-conjugated length 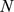 for PTh (yellow circles), PITN (blue squares), and PATV (purple diamonds). The bandgap of ITN is 2 eV lower than Th because of the energy level splitting between Th and Bz.

In systems such as PITN with aromatic rings fused perpendicular to the conjugated pathway, the monomers themselves have lower bandgaps simply due to energy level splitting. Fig. 5 shows the energy level splitting for poly-isothianaphthene (PITN), [55] depicted as a combination of Th and benzene (Bz) orbitals sharing two carbons. Although the ITN monomer has a bandgap nearly 2 eV smaller than the Th monomer, the bandgap of PITN (Fig. 5, blue squares) is only 0.8 eV smaller than PTh (Fig. 5, yellow circles), indicating that the highly aromatic ITN unit actually has a smaller electronic overlap with neighboring units. In other words, the lower gap of the polymer is due to the lower starting point of the monomer overcoming a weaker polymerization effect. This effect is exacerbated in poly-(9,10-anthrylene vinylene) (PATV), which uses anthracene as its monomer with three fused rings perpendicular to the chain direction. Although the ATV monomer has a bandgap even lower than ITN, polymerization does not significantly lower the bandgap any further (Fig. 5, purple diamonds). Using the optical gap slope with respect to the chain length of PITN and PATV, we obtain the 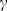 parameters of 0.75 and 0.2 for perpendicular fused rings of size two and three, respectively.

In order to decrease the bandgaps for polymers consisting of perpendicular fused rings we must to enhance the electron hopping between the connected monomers. This enhancement can be achieved by introducing parallel fused ring monomers between each perpendicular fused ring monomer to form copolymers, examples of which are discussed in the copolymers section.

### Copolymers

**Figure 6:**
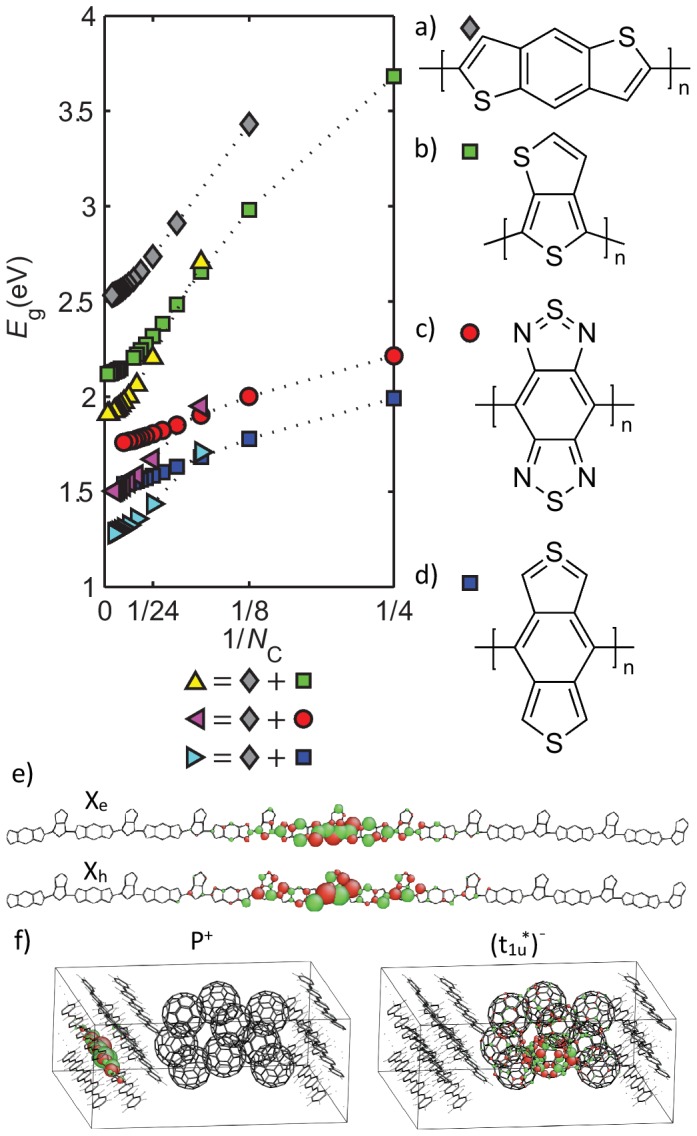
Bandgaps of copolymers and their parent regioregular polymers, including BDT of parallel fused rings (gray diamonds), Tt (green squares), BBTD (red circles), and BBT (blue squares). 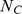
 is the number of carbon atoms in the conjugated pathway. The yellow up-triangles, magenta left-triangles, and cyan right-triangles are copolymers of BDT with Tt, BBTD, and BBT, respectively. Copolymers show steep bandgap reductions via polymerization. (e) Excitons in copolymers do not show spontaneous charge separations, where the electron 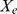 and hole 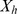 states extend over both the BDT and Tt units. (f) In contrast, spontaneous charge separations occur at the bulk heterojunction interfaces, leaving behind a hole polaron state 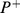 in the polymer phase and an electron state 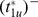 in the 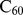 phase.

Copolymers of donor-acceptor units have been leading the way in improving organic PV efficiency from 5% to 9%. [56] Despite using the same parameters as used on the much simpler structures, the copolymers group show agreement (depicted in Fig. 2d) with experimental values of 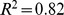, 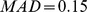 eV, and 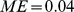 eV, on par with the other gropus in this study. The calculations demonstrate that, as shown in Fig. 6, copolymers (triangles) with small conjugation lengths 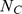 do not necessarily have lower bandgaps than their parent regioregular polymers (circles or squares) of the same 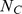, where 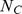 is the number of carbon atoms along the conjugated pathway. The lower bandgaps in longer chain lengths are due to stronger polymerization effects, through the parallel fused rings that strengthen electronic communications between perpendicularly fused units. This effect is valid even when combining two typical acceptors (electron poor units), such as benzo[1,2-b:4,3-b']dithiophene (BDT) (Fig. 6a, gray diamonds) and benzobisthiadiazole (BBTD) (Fig. 6c, red circles), for which case a bandgap of 1.4 eV is predicted (magenta triangles). Furthermore, our calculations clearly show that there are no real electronic distinctions between donor and acceptor units in co-polymers, since upon photoexcitations the localized excitonic electron- and hole-wavefunctions span over both the Tt donor (Fig. 6b, green squares) and BDT acceptor units along the same 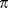-conjugated polymer backbone as shown in Fig. 6e. Our calculations also indicate that these copolymers do not form two separated domain walls of irrational charges. [57] As a sharp contrast, the electron and hole states can be spontaneously separated across the fullerene-polymer heterojunctions through 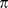-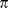 stacking (Fig. 6f). [25] With this in mind, we further propose to replace the N atoms in BBTD with carbons (Fig. 6d, blue squares) to form a copolymer that reaches the 1.2 eV target (cyan triangles). When taking into account that favorable 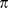-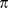 stacking effects can lower the bandgap by a further 0.4 eV, [25] our calculations suggest that an optical bandgap as low as 0.8 eV may be possible, making it a candidate for the long wavelength absorbing materials in tandem OPV cells.

### Polycyclic Aromatic Hydrocarbons

**Figure 7:**
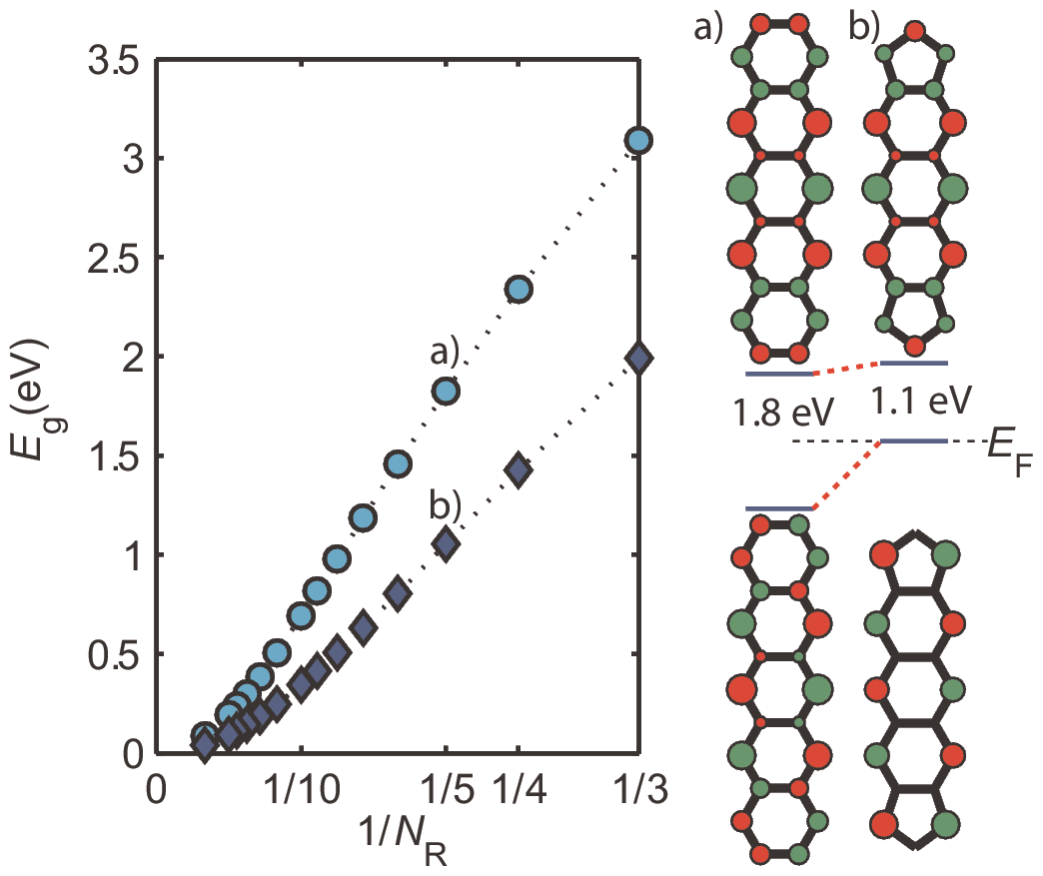
Bandgap comparison between polyacenes and thiophene capped polyacenes as a function of the number of aromatic rings 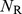. The latter have lower bandgaps for comparable molecular sizes. As an example, the HOMO and LUMO levels of pentacene (a) is compared with thiophene capped anthracene (b), showing how the reduction in the wavefunction of the shared carbon sites on the HOMO is mostly responsible for the bandgap differences.

PAHs are essentially flakes of graphene, the Fermi energy of which varies linearly with wavevector through the Dirac point without undergoing the Peierls dimerization [58], [59]. In short polyacenes, nonacene (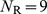) being the longest ever synthesized to date [60], [61], the inevitable edge dimerization effects lead to the characteristic bond-centered HOMO and LUMO phase patterns near the edges (Fig. 7a) that open their bandgaps. For pentacene (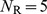), we find 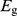 = 1.8 eV. For much longer polyacenes, the ground state may be an open shell singlet diradical [62], so our discussions will be limited to the shorter molecules. The agreement with experimental values is shown in Fig. 2e, with 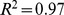, 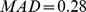 eV, 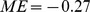 eV. The relatively high 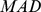 value is due to the high 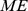 value, a consequence of choosing 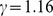 for all benzene-type rings to simplify usage of the model. The trends are nevertheless correct, demonstrated by the 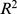 near unity.

In order to reduce the optical gap of polyacenes, one can create nodes on the 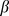 sites (the carbons shared by the rings) by fusing 5 membered rings to the ends. Common in all 5-membered aromatic rings [23], the HOMO level of Th (Fig. 3, V band) is bonding between 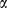-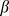 sites and anti-bonding between 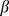-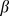 sites and has nodes on S sites. When fusing Th to anthracene of 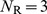 (Fig. 7b), the two 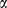-C's of Th naturally extend the non-bonding wavefunction phase pattern of polyacenes to new S edges and leave nodes precisely on 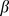 sites. This effectively shifts the HOMO level up to the Dirac point and reduces 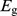 from 1.8 to 1.1 eV. Alternatively, one may substitute the 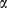-C's of Th with N's to form a similar structure to the thiadiazole units in BBTD (Fig. 6c). This gives an 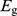 of 1.21 eV.

## Conclusion

The accurate prediction of the optical bandgap of a generic materials system will remain as a grand challenge in condensed matter theory. Working towards that goal, we demonstrate a model Hamiltonian for virtually all the major types of 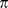-conjugated systems in which electron correlations are renormalized into quasi-particles through the universal, and therefore transferable, electron-phonon coupling parameters [11], [19], [23], [24]. With excellent agreement to their corresponding experimental measurements over a comprehensive data set, our results indicate that residual correlation effects beyond such universal electron-phonon couplings are not essential as far as the optical bandgaps are concerned. Similarly, and corroborated by the fused thiophene series, we find the lack of explicit planarity to have a negligeable impact on the accuracy of the model. The aSSH Hamiltonian is an accurate, transferable, and efficient computational tool that provides the quantitative description of structure-property relationships afforded by quantum mechanical theories.

From our calculations, the strategies of planarization or simple substitutions did not lead to the target optical gaps 1.2 eV. To reach the desired gaps, we suggest a copolymer of parallel and perpendicular monomers (both benzodithiophenes), which we find in general to be the best strategy by taking advantage of both the lower monomer gaps of perpendicular units and the enhanced electronic communication of the parallel ones. We also propose a small molecule oligomer (a thiophene capped anthracene) that arises from our analysis of PAHs, in particular of pentacene, with the aSSH model giving insights into the electronic structure of the molecules and how to modify them for lower gaps. With these examples, we put forth the aSSH model as an in-silico design tool to guide the search for optimized organic electronics.

## Supporting Information

Figure S1 **Skeletal formulae of simple ring systems.** Simple ring systems consist of monomers that are single aromatic rings that may contain single atoms or non-conjugated rings attached.
(PDF)

Figure S2 **Skeletal formulae of parallel fused ring systems.** Parallel fused ring systems consist of monomers wherein two or more aromatic rings are fused and all rings are part of the main conjugated pathway.
(PDF)

Figure S3 **Skeletal formulae of perpendicular fused ring systems.** Perpendicular fused ring systems consist of monomers wherein two or more aromatic rings are fused, but only one of the rings is connected to the main conjugated pathway.
(PDF)

Figure S4 **Skeletal formulae of polycyclic aromatic hydrocarbons and derivatives.** Polycyclic aromatic hydrocarbons are made entirely of fused aromatic carbon rings, while derivatives include simple heteroatom substitutions for hydrogen.
(PDF)

Figure S5 **Skeletal formulae of copolymers.** Copolymers refer to any system made of more than one type of monomer.
(PDF)

Figure S6
**Skeletal formulae of** 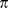 **-** 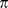 **stacking systems.** The 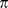-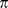 stacking systems consist of multiple separate oligomers necessitating an inter-chain interaction term.(PDF)

Table S1 **Experimental and aSSH calculated optical gaps for simple ring polymers.**
(PDF)

Table S2 **Experimental and aSSH calculated optical gaps for parallel fused ring systems.**
(PDF)

Table S3 **Experimental and aSSH calculated optical gaps for perpendicular fused ring systems.**
(PDF)

Table S4 **Experimental and aSSH calculated optical gaps for polycyclic aromatic hydrocarbons and derivatives.**
(PDF)

Table S5 **Experimental and aSSH calculated optical gaps for copolymers.**
(PDF)

Table S6
**Experimental and aSSH calculated optical gaps for** 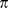 **-** 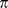 **stacking systems.**
(PDF)

References S1 **References for data contained in Tables S1–6.** Although some references for the Supporting Information may be the same as for the main article, their reference numbers may not be the same.
(PDF)
