# Supplementary figures and images for: Structure and Optical Bandgap Relationship of π-Conjugated Systems
Source: PLoS One. 2014 Jan 31;9(1):e86370. doi: 10.1371/journal.pone.0086370 (PMC3908919; doi:10.1371/journal.pone.0086370)

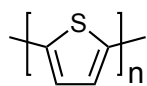

PTh

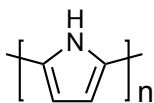

PPy

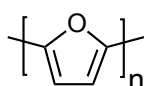

PFu

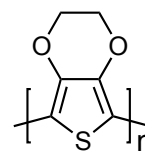

PEDOT

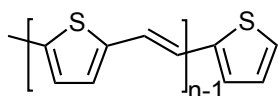

PTV

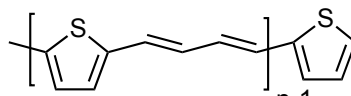

PTW

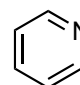

Pyridine

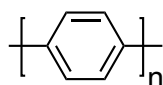

PPP

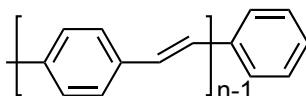

PPV

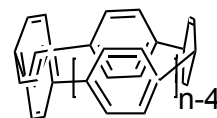

PPP nanohoop

Supplement: Figure S1 — Skeletal formulae of simple ring systems. Simple ring systems consist of monomers that are single aromatic rings that may contain single atoms or non-conjugated rings attached. (PDF) [file pone.0086370.s001.pdf]

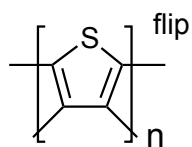

PTA

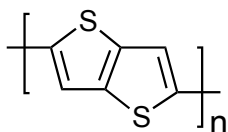

PT32bT

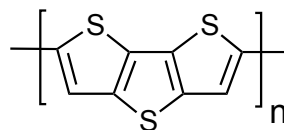

PT32b23dT

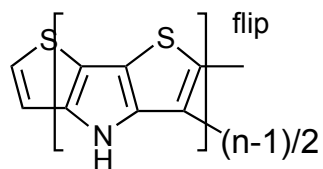

NBTT

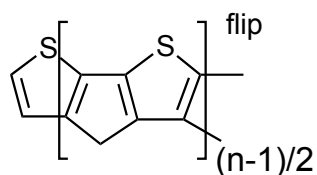

CDT

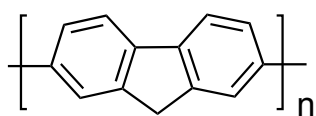

PFO

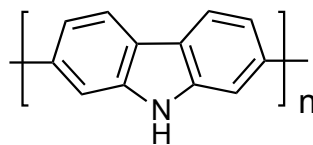

PCZ

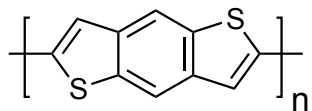

BDT

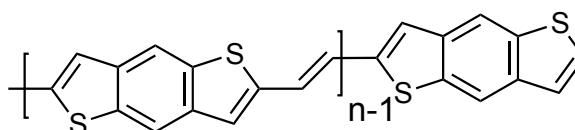

BDTV

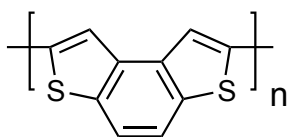

m-BDT

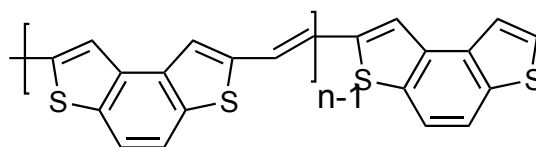

m-BDTV

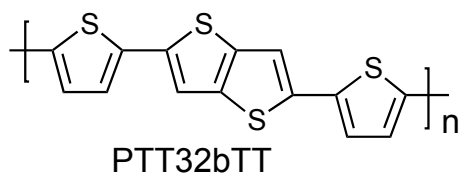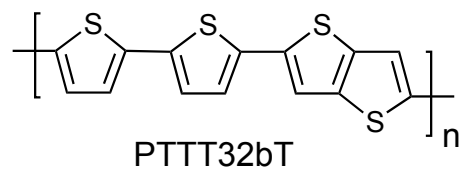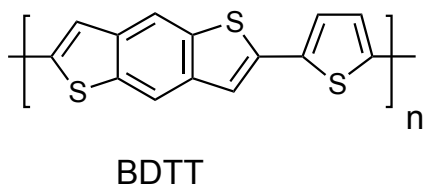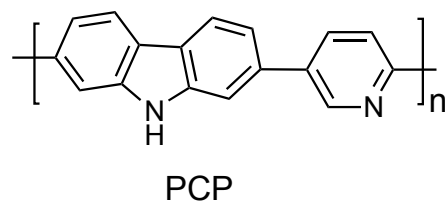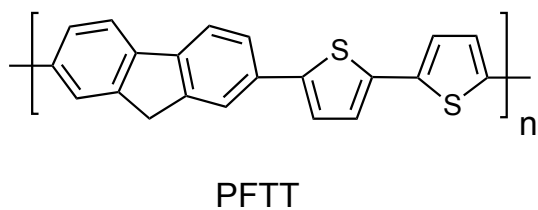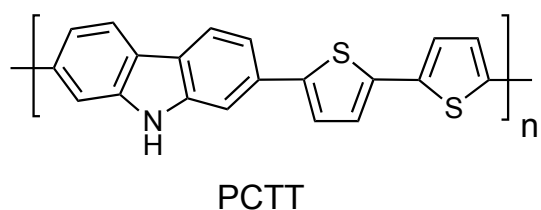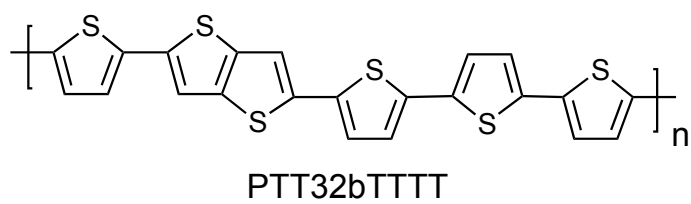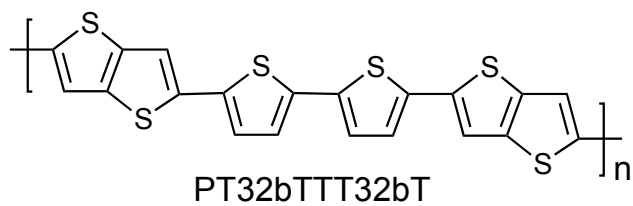

Supplement: Figure S2 — Skeletal formulae of parallel fused ring systems. Parallel fused ring systems consist of monomers wherein two or more aromatic rings are fused and all rings are part of the main conjugated pathway. (PDF) [file pone.0086370.s002.pdf]

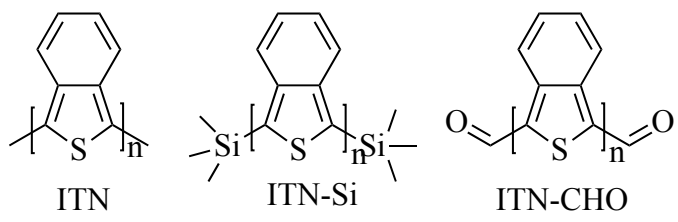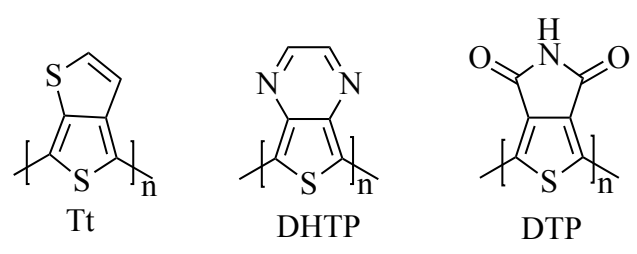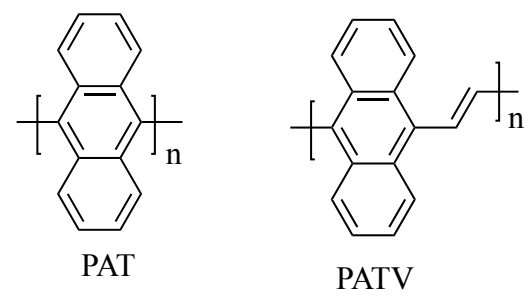

Supplement: Figure S3 — Skeletal formulae of perpendicular fused ring systems. Perpendicular fused ring systems consist of monomers wherein two or more aromatic rings are fused, but only one of the rings is connected to the main conjugated pathway. (PDF) [file pone.0086370.s003.pdf]

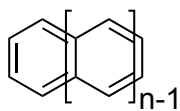

PAcene

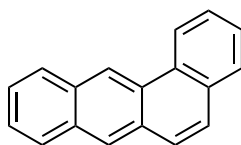

BA

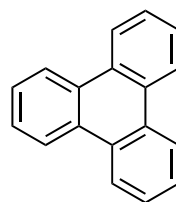

TP

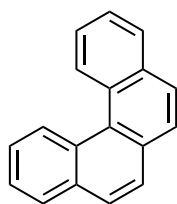

BP

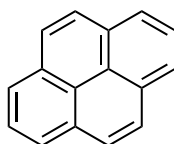

pyrene

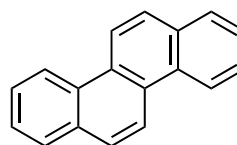

Chr

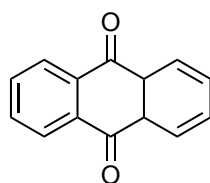

AQ

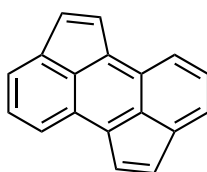

CPAA

Supplement: Figure S4 — Skeletal formulae of polycyclic aromatic hydrocarbons and derivatives. Polycyclic aromatic hydrocarbons are made entirely of fused aromatic carbon rings, while derivatives include simple heteroatom substitutions for hydrogen. (PDF) [file pone.0086370.s004.pdf]

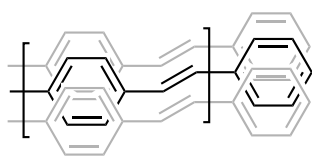

PPVs

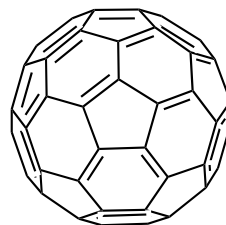

C<sub>60</sub>

Supplement: Figure S6 — Skeletal formulae of - stacking systems. The - stacking systems consist of multiple separate oligomers necessitating an inter-chain interaction term. (PDF) [file pone.0086370.s006.pdf]
