# Supplementary material for: Structure and Optical Bandgap Relationship of π-Conjugated Systems
Source: PLoS One. 2014 Jan 31;9(1):e86370. doi: 10.1371/journal.pone.0086370 (PMC3908919; doi:10.1371/journal.pone.0086370)
Supplement: Figure S5 — Skeletal formulae of copolymers. Copolymers refer to any system made of more than one type of monomer. (PDF) [file pone.0086370.s005.pdf]

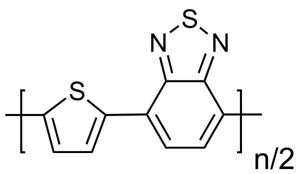

TBTD

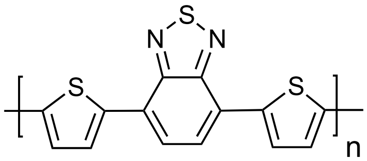

DTBTD

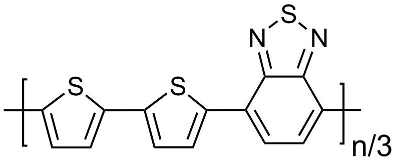

BTBTD

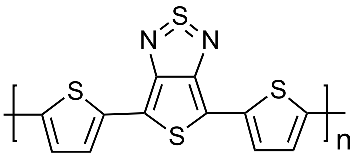

DTTTD

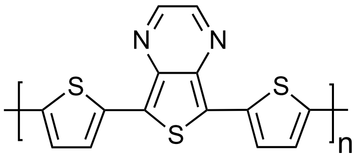

DTTP

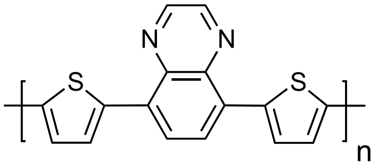

DTQU

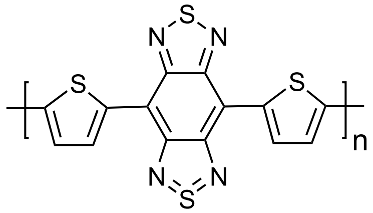

DTBBTD

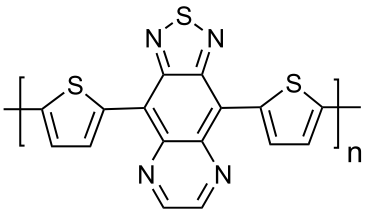

DTTDQ

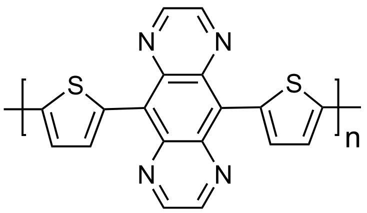

DTPQU

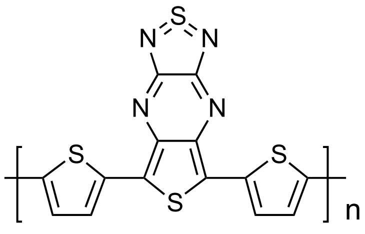

DTTPT

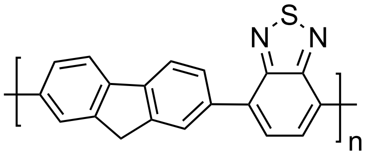

PFTBT

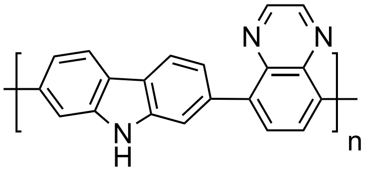

PCQX

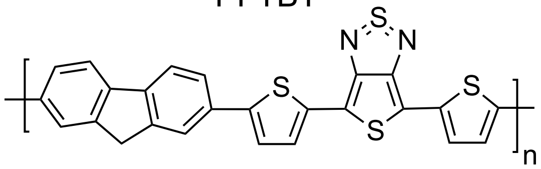

PFTTD

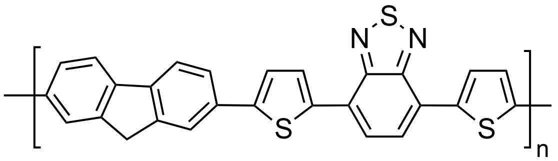

PFDTBT

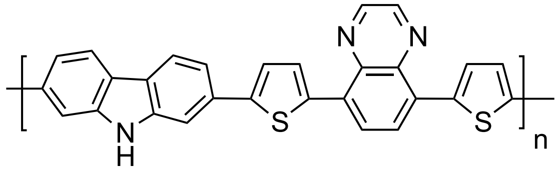

PCDTQX

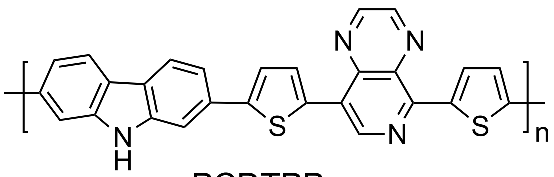

PCDTPP

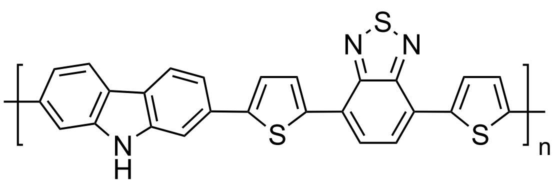

PCDTBT

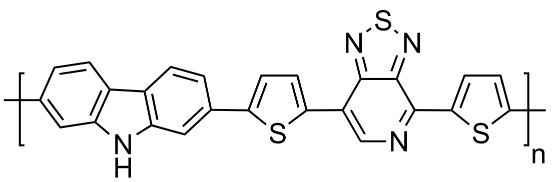

PCDTPT

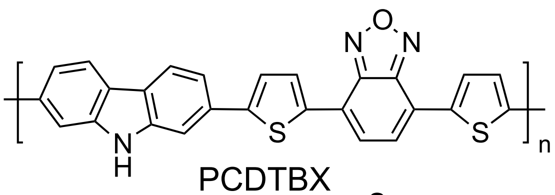

PCDTBX

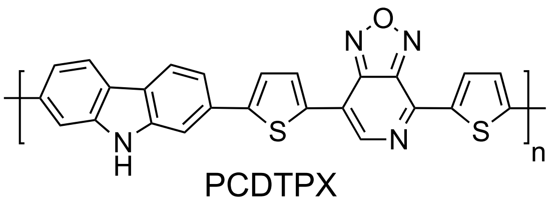

PCDTPX

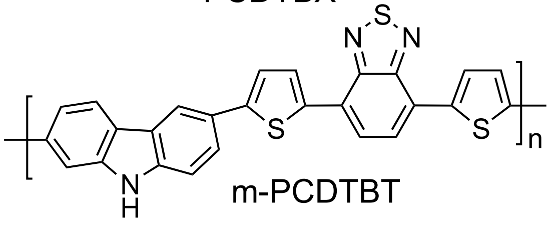

m-PCDTBT

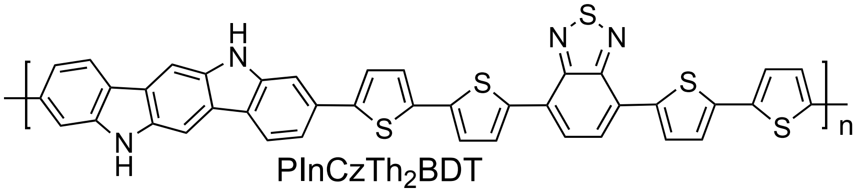

PInCzTh$_2$BDT

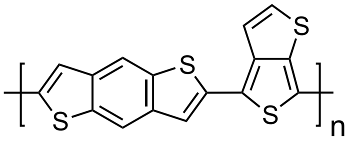

PTB

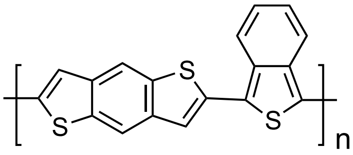

PBDTITN

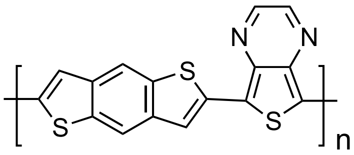

PBDTTP

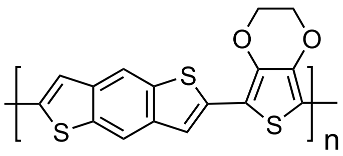

PBDTEDT

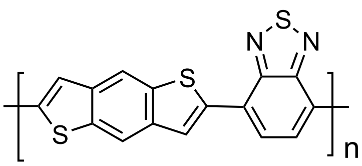

PBDTBT

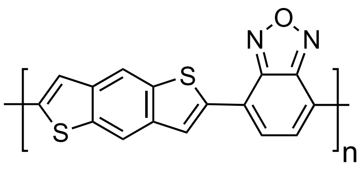

PBDTBX

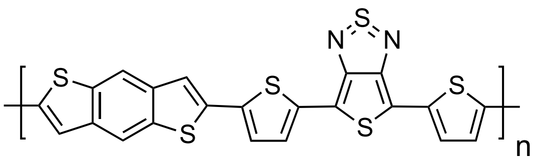

PBDTTT

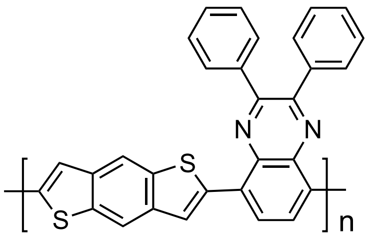

PBDTTQUDB

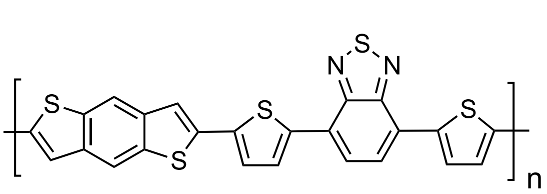

PBDTDTBT

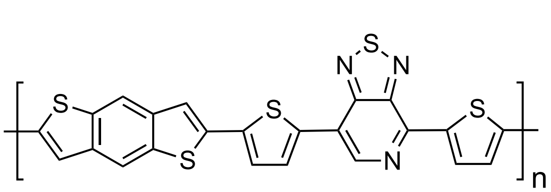

PBDTDTPT

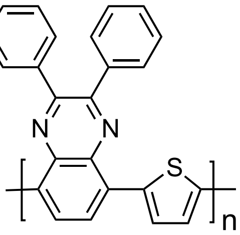

PTQUDB

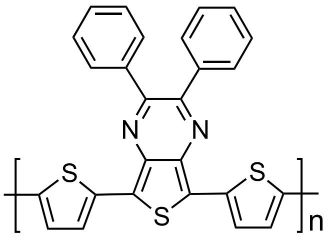

PDTTPDB

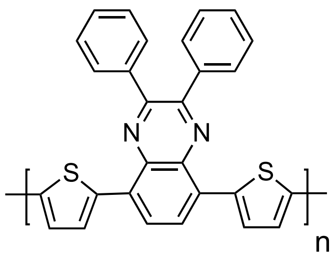

PDTQUDB

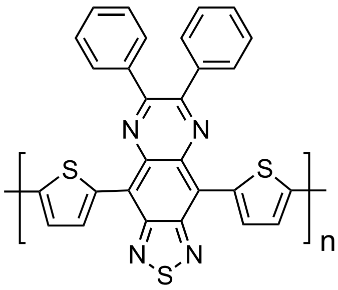

PDTDBTDQ

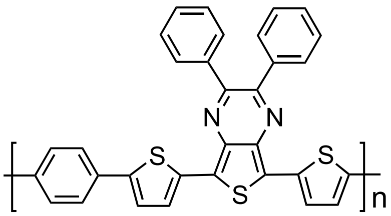

PBDTTPDB

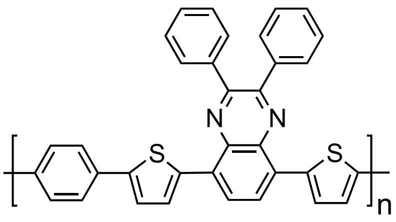

PBDTQUDB

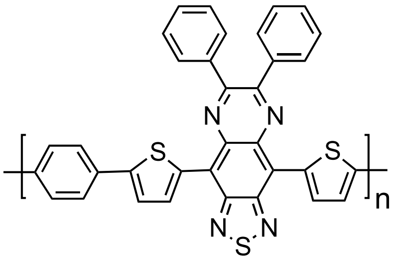

PBDTDBTDQ

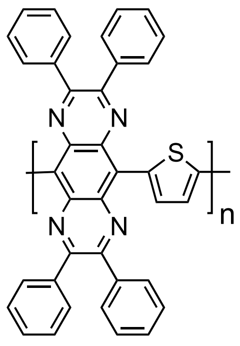

PTPQUTB

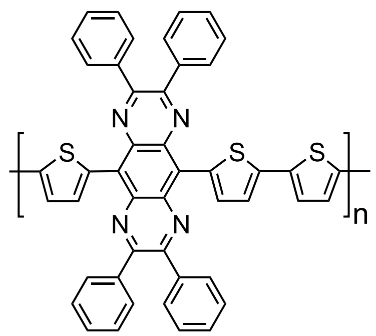

PTDTPQUTB

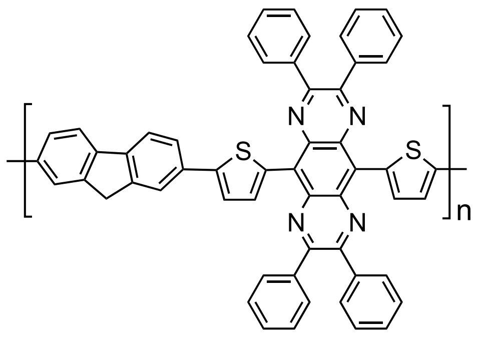

PFDTPQUTB

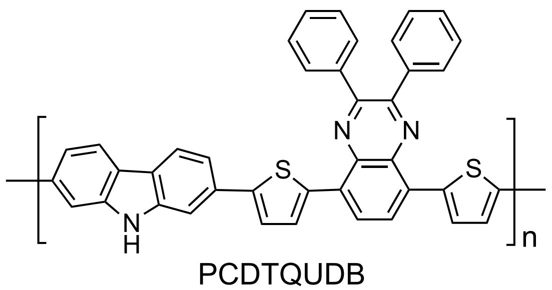

PCDTQUDB

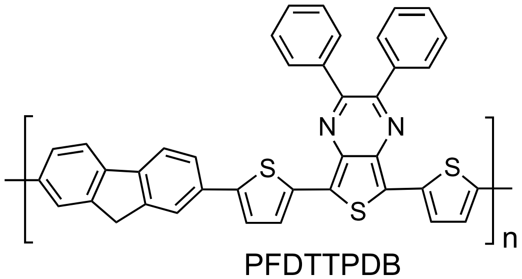

PFDTTPDB

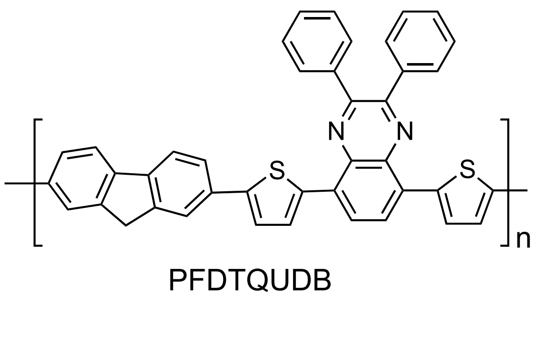

PFDTQUDB

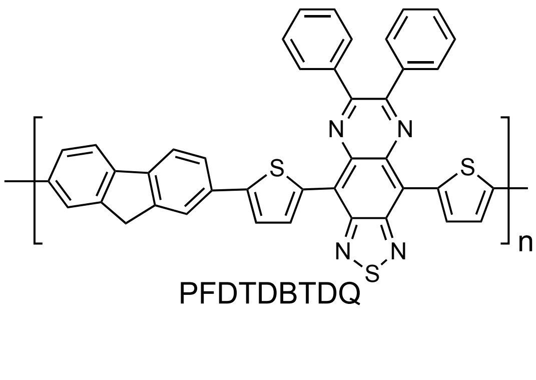

PFDTDBTDQ

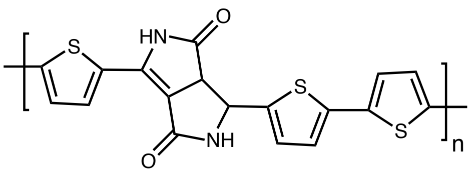

PDPP3T

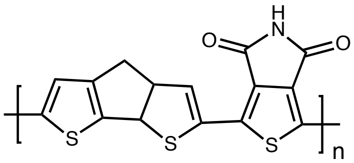

PFTPD

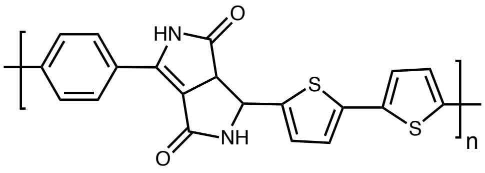

PDPPTPT

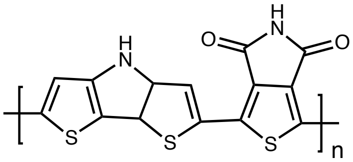

PCTPD

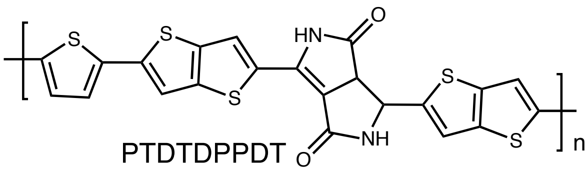

PTDTDPPDT

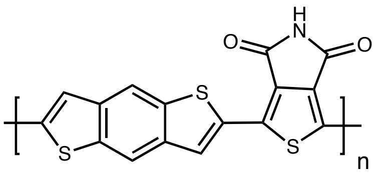

PBDTTPD

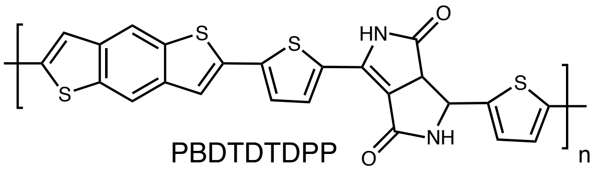

PBDTDTDPP

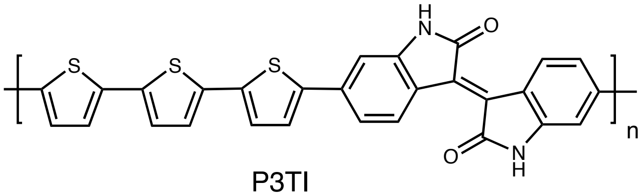

P3TI
