# Supplementary material for: Structure and Optical Bandgap Relationship of π-Conjugated Systems
Source: PLoS One. 2014 Jan 31;9(1):e86370. doi: 10.1371/journal.pone.0086370 (PMC3908919; doi:10.1371/journal.pone.0086370)
Supplement: Table S1 — Experimental and aSSH calculated optical gaps for simple ring polymers. (PDF) [file pone.0086370.s007.pdf]

| | Ref. | $n$ | Exp. $E_g$ (eV) | aSSH $E_g$ (eV) |
|---|---|---|---|---|
| PEDOT | S1[1] | 2 | 3.56 | 3.50 |
| PEDOT | S1[2] | 2 | 3.71 | 3.50 |
| PEDOT | S1[1] | 3 | 3.00 | 2.97 |
| PEDOT | S1[2] | 3 | 3.03 | 2.97 |
| PEDOT | S1[1] | 4 | 2.70 | 2.70 |
| PEDOT | S1[2] | 4 | 2.71 | 2.70 |
| PEDOT | S1[2] | 5 | 2.49 | 2.55 |
| PEDOT | S1[3] | 20 | 1.90 | 2.18 |
| PFu | S1[4] | 1 | 5.90 | 5.36 |
| PFu | S1[4] | 2 | 4.11 | 3.95 |
| PFu | S1[4] | 3 | 3.58 | 3.37 |
| PFu | S1[4] | 4 | 3.25 | 3.18 |
| PFu | S1[5] | 5 | 3.02 | 3.03 |
| PFu | S1[5] | 6 | 2.89 | 2.93 |
| PFu | S1[5] | 7 | 2.81 | 2.87 |
| PFu | S1[5] | 8 | 2.76 | 2.83 |
| PFu | S1[5] | 9 | 2.72 | 2.80 |
| PPP | S1[6] | 1 | 5.90 | 5.92 |
| PPP | S1[6] | 2 | 4.96 | 4.65 |
| PPP | S1[6] | 3 | 4.43 | 4.15 |
| PPP | S1[6] | 4 | 4.13 | 3.90 |
| PPP | S1[6] | 5 | 4.00 | 3.76 |
| PPP nanohoop | S1[7] | 5 | 3.66 | 3.42 |
| PPP nanohoop | S1[7] | 8 | 3.69 | 3.41 |
| PPP nanohoop | S1[7] | 14 | 3.67 | 3.41 |
| PPV | S1[8] | 2 | 3.60 | 3.75 |
| PPV | S1[8] | 3 | 3.01 | 3.25 |
| PPV | S1[8] | 4 | 2.90 | 3.04 |
| PPV | S1[8] | 5 | 2.75 | 2.93 |
| PPy | S1[6] | 1 | 5.96 | 5.67 |
| PPy | S1[9] | 1 | 5.96 | 5.67 |
| PPy | S1[6] | 2 | 4.35 | 4.23 |
| PPy | S1[9] | 2 | 4.49 | 4.23 |
| PPy | S1[6] | 3 | 3.59 | 3.76 |
| PPy | S1[9] | 3 | 3.91 | 3.76 |
| PPy | S1[9] | 5 | 3.38 | 3.39 |
| PPy | S1[9] | 7 | 3.25 | 3.25 |
| PTh | S1[10] | 1 | 5.37 | 5.24 |
| PTh | S1[11] | 1 | 5.10 | 5.24 |

*Continued on next page*

Table S1 – *Continued from previous page*

| | Ref. | $n$ | Exp. $E_g$ (eV) | aSSH $E_g$ (eV) |
|---|---|---|---|---|
| PTh | S1[10] | 2 | 4.09 | 3.87 |
| PTh | S1[12] | 2 | 4.05 | 3.87 |
| PTh | S1[11] | 2 | 4.11 | 3.87 |
| PTh | S1[10] | 3 | 3.50 | 3.37 |
| PTh | S1[12] | 3 | 3.49 | 3.37 |
| PTh | S1[11] | 3 | 3.50 | 3.37 |
| PTh | S1[13] | 4 | 3.27 | 3.12 |
| PTh | S1[10] | 4 | 3.16 | 3.12 |
| PTh | S1[12] | 4 | 3.16 | 3.12 |
| PTh | S1[11] | 4 | 3.18 | 3.12 |
| PTh | S1[10] | 5 | 2.97 | 2.98 |
| PTh | S1[12] | 5 | 2.99 | 2.98 |
| PTh | S1[11] | 5 | 2.98 | 2.98 |
| PTh | S1[10] | 6 | 2.84 | 2.89 |
| PTh | S1[12] | 6 | 2.85 | 2.89 |
| PTh | S1[11] | 6 | 2.87 | 2.89 |
| PTh | S1[10] | 7 | 2.81 | 2.83 |
| PTh | S1[13] | 8 | 2.82 | 2.79 |
| PTh | S1[13] | 12 | 2.72 | 2.71 |
| PTh | S1[13] | 16 | 2.67 | 2.68 |
| PTV | S1[14] | 3 | 2.79 | 2.61 |
| PTV | S1[14] | 4 | 2.53 | 2.40 |
| PTV | S1[14] | 5 | 2.38 | 2.29 |
| PTVV | S1[14] | 2 | 3.25 | 2.63 |
| PTVV | S1[14] | 3 | 2.66 | 2.22 |
| PTVV | S1[14] | 4 | 2.38 | 2.05 |
| Pyridine | S1[15] | 1 | 4.99 | 4.99 |
