# Supplementary material for: Structure and Optical Bandgap Relationship of π-Conjugated Systems
Source: PLoS One. 2014 Jan 31;9(1):e86370. doi: 10.1371/journal.pone.0086370 (PMC3908919; doi:10.1371/journal.pone.0086370)
Supplement: Table S2 — Experimental and aSSH calculated optical gaps for parallel fused ring systems. (PDF) [file pone.0086370.s008.pdf]

	Ref.	n	Exp. E_g (eV)	aSSH E_g (eV)
BDT	S1[16]	1	3.58	3.34
BDT	S1[16]	4	2.65	2.59
BDTT	S1[17]	73	2.42	2.51
BDTV	S1[17]	115	2.42	2.20
CDT	S1[18]	3	3.99	3.87
CDT	S1[19]	5	3.34	3.37
m-BDT	S1[20]	2	3.08	3.08
m-BDT	S1[20]	3	2.91	2.89
m-BDT	S1[20]	4	2.82	2.81
m-BDT	S1[20]	5	2.79	2.76
m-BDTV	S1[20]	2	2.89	2.80
m-BDTV	S1[20]	3	2.68	2.63
m-BDTV	S1[20]	4	2.60	2.56
m-BDTV	S1[20]	5	2.56	2.53
NBTT	S1[21]	5	3.28	3.63
PCP	S1[22]	11	3.51	3.15
PCTT	S1[23]	31	2.74	2.85
PCZ	S1[24]	69	3.88	3.55
PCZ	S1[23]	167	3.18	3.55
PFO	S1[25]	14	3.31	3.42
PFTT	S1[26]	20	3.13	2.71
PT32b23dTT	S1[27]	1	3.53	3.41
PT32bT	S1[28]	1	4.43	4.33
PT32bT	S1[29]	2	3.55	3.31
PT32bT	S1[30]	3	3.10	2.95
PT32bT	S1[28]	10	2.78	2.55
PT32bTTT32bT	S1[30]	1	2.99	2.91
PTA	S1[29]	5	3.48	3.49
PTA	S1[29]	7	3.13	3.22
PTT32bTT	S1[29]	1	3.35	3.17
PTT32bTT	S1[31]	20	2.66	2.58
PTT32bTTTT	S1[30]	1	2.91	2.89
PTTT32bT	S1[29]	1	3.35	3.20
