# Supplementary material for: Structure and Optical Bandgap Relationship of π-Conjugated Systems
Source: PLoS One. 2014 Jan 31;9(1):e86370. doi: 10.1371/journal.pone.0086370 (PMC3908919; doi:10.1371/journal.pone.0086370)
Supplement: Table S3 — Experimental and aSSH calculated optical gaps for perpendicular fused ring systems. (PDF) [file pone.0086370.s009.pdf]

	Ref.	n	Exp. E_g (eV)	aSSH E_g (eV)
DHTP	S1[32]	12	1.42	1.91
DTP	S1[33]	13	2.76	2.76
ITN-CHO	S1[34]	1	3.03	2.53
ITN-CHO	S1[34]	2	2.59	2.23
ITN-CHO	S1[34]	3	2.31	2.08
ITN-Si	S1[35]	1	3.58	3.28
ITN-Si	S1[35]	2	2.99	2.62
ITN-Si	S1[35]	3	2.65	2.32
ITN-Si	S1[35]	4	2.46	2.16
PAT	S1[36]	2	3.20	2.92
PAT	S1[36]	3	3.14	2.89
PATV	S1[37]	2	3.08	2.98
PATV	S1[37]	3	2.98	2.95
PATV	S1[37]	4	2.89	2.95
PATV	S1[37]	5	2.83	2.94
PATV	S1[37]	6	2.78	2.94
PATV	S1[37]	7	2.78	2.94
Tt	S1[38]	18	2.02	2.15
Tt	S1[38]	21	1.95	2.14
Tt	S1[38]	21	2.06	2.14
Tt	S1[38]	24	2.11	2.14
