# Supplementary material for: Structure and Optical Bandgap Relationship of π-Conjugated Systems
Source: PLoS One. 2014 Jan 31;9(1):e86370. doi: 10.1371/journal.pone.0086370 (PMC3908919; doi:10.1371/journal.pone.0086370)
Supplement: Table S4 — Experimental and aSSH calculated optical gaps for polycyclic aromatic hydrocarbons and derivatives. (PDF) [file pone.0086370.s010.pdf]

	Ref.	n	Exp. E_g (eV)	aSSH E_g (eV)
AQ	S1[39]	–	3.82	3.11
BA	S1[40]	–	3.26	3.17
BP	S1[41]	–	3.95	3.66
Chr	S1[40]	–	3.75	3.51
CPAA	S1[42]	–	2.22	2.29
PAcene	S1[43]	2	4.55	4.11
PAcene	S1[44]	3	3.28	2.99
PAcene	S1[45]	4	2.61	2.26
PAcene	S1[46]	5	2.12	1.76
PAcene	S1[47]	6	1.57	1.41
PAcene	S1[47]	7	1.36	1.15
pyrene	S1[40]	–	3.35	3.12
pyrene	S1[48]	–	3.32	3.12
TP	S1[41]	–	4.37	4.16
