# Supplementary material for: Structure and Optical Bandgap Relationship of π-Conjugated Systems
Source: PLoS One. 2014 Jan 31;9(1):e86370. doi: 10.1371/journal.pone.0086370 (PMC3908919; doi:10.1371/journal.pone.0086370)
Supplement: Table S5 — Experimental and aSSH calculated optical gaps for copolymers. (PDF) [file pone.0086370.s011.pdf]

	Ref.	n	Exp. E_g (eV)	aSSH E_g (eV)
BTBTD	S1[49]	3	2.89	2.77
BTBTD	S1[49]	5	2.47	2.46
DTBBTD	S1[50]	1	1.77	1.54
DTBTD	S1[51]	1	2.66	2.70
DTBTD	S1[50]	1	2.79	2.70
DTBTD	S1[49]	1	2.78	2.70
DTBTD	S1[49]	2	2.38	2.40
DTPQU	S1[51]	1	2.53	2.16
DTQU	S1[51]	1	3.06	2.80
DTTDQ	S1[51]	1	2.05	1.90
DTTP	S1[51]	1	2.35	2.33
DTTPT	S1[52]	1	1.25	1.34
DTTTD	S1[53]	1	2.01	2.03
m-PCDTBT	S1[54]	3	2.35	2.53
P3TI	S1[55]	36	1.88	1.82
PBDTBT	S1[17]	27	2.10	2.26
PBDTBX	S1[56]	76	1.90	1.98
PBDTDBTDQ	S1[57]	13	1.28	1.62
PBDTDTBT	S1[58]	23	1.91[a]	2.29
PBDTDTBT	S1[59]	35	2.03	2.29
PBDTDTDPP	S1[60]	30	1.64	1.54
PBDTDTPT	S1[61]	100	1.68	2.06
PBDTEDT	S1[17]	42	2.33	2.34
PBDTITN	S1[62]	22	2.00	1.84
PBDTQUDB	S1[63]	5	2.16	2.27
PBDTTP	S1[17]	50	1.59	1.88
PBDTTPD	S1[64]	30	2.04	2.45
PBDTTPDB	S1[65]	6	1.79	1.96
PBDTTQUDB	S1[17]	18	2.07	1.96
PBDTTT	S1[66]	7	1.54	1.76
PCDTBT	S1[67]	20	2.30	2.47
PCDTBX	S1[67]	20	2.28	2.23
PCDTPP	S1[67]	16	2.30	2.22
PCDTPT	S1[67]	6	2.19	2.25
PCDTPX	S1[67]	7	2.16	2.03
PCDTQUDB	S1[68]	11	2.38	2.35
PCDTQX	S1[67]	13	2.46	2.55
PCQX	S1[22]	17	3.09	2.93
PCTPD	S1[69]	16	1.92	2.38

Continued on next page

Table S5 – *Continued from previous page*

	Ref.	n	Exp. E_g (eV)	aSSH E_g (eV)
PDPP3T	S1[70]	65	1.45	1.51
PDPPTPT	S1[71]	13	1.59	1.52
PDTDBTDQ	S1[72]	331	1.53	1.41
PDTQUDB	S1[72]	14	2.48	2.02
PDTTPDB	S1[72]	35	1.55	1.77
PFDTBT	S1[73]	7	2.26	2.44
PFDTDBTDQ	S1[57]	4	1.59	1.70
PFDTPQUTB	S1[57]	13	1.73	1.83
PFDTQUDB	S1[63]	10	2.29	2.34
PFDTTPDB	S1[57]	13	1.88[a]	2.01
PFTBT	S1[74]	20	2.40	2.57
PFTBT	S1[75]	20	2.66	2.57
PFTPD	S1[69]	24	1.90	2.41
PFTTD	S1[76]	6	1.72	1.76
PFTTD	S1[77]	35	1.74	1.74
PInCzTTBTTT	S1[78]	16	2.31	2.39
PTB	S1[79]	23	1.84	1.96
PTDTDPPDT	S1[80]	18	1.45	1.42
PTDTPQUTB	S1[81]	98	1.67	1.52
PTPQUTB	S1[81]	88	1.45	1.45
PTQUDB	S1[82]	67	2.03	2.07
TBTD	S1[49]	2	3.18	3.01

Note:
a: Polymer films
