# Supplementary material for: Structure and Optical Bandgap Relationship of π-Conjugated Systems
Source: PLoS One. 2014 Jan 31;9(1):e86370. doi: 10.1371/journal.pone.0086370 (PMC3908919; doi:10.1371/journal.pone.0086370)
Supplement: Table S6 — Experimental and aSSH calculated optical gaps for - stacking systems. (PDF) [file pone.0086370.s012.pdf]

	Ref.	$n \times N$	Exp. E_g (eV)	aSSH E_g (eV)
C_{60}	S1[83]	1×9	2.55^a	$2.70^{a,c}$
C_{60}	S1[84]	1×9	2.32^b	$2.28^{b,c}$
PPVs	S1[85]	5×12	2.50	2.48^c

Note:
a: Optical bandgap of C_{60}
b: HOMO-LUMO bandgap of C_{60}
c: Reference S1[86]
