# Supplementary material for: Structure and Optical Bandgap Relationship of π-Conjugated Systems
Source: PLoS One. 2014 Jan 31;9(1):e86370. doi: 10.1371/journal.pone.0086370 (PMC3908919; doi:10.1371/journal.pone.0086370)
Supplement: References S1 — References for data contained in Tables S1–6. Although some references for the Supporting Information may be the same as for the main article, their reference numbers may not be the same. (PDF) [file pone.0086370.s013.pdf]

# References S1


1. Turbiez M, Frère P, Roncali J (2003) Stable and soluble oligo(3,4-ethylene-dioxythiophene)s end-capped with alkyl chains. Journal of Organic Chemistry 68: 5357–5360.

2. Wasserberg D, Meskers SCJ, Janssen RAJ, Mena-Osteritz E, Buerle P (2006) High-Resolution electronic spectra of ethylenedioxythiophene oligomers. Journal of the American Chemical Society 128: 17007–17017.

3. Roncali J, Blanchard P, Frère P (2005) 3,4-Ethylenedioxythiophene (EDOT) as a versatile building block for advanced functional $\pi$-conjugated systems. Journal of Materials Science 15: 1589–1610.

4. de Melo JS, Elisei F, Gartner C, Aloisi GG, Becker RS (2000) Comprehensive investigation of the photophysical behavior of oligopolyfurans. The Journal of Physical Chemistry A 104: 6907–6911.

5. Gidron O, Diskin-Posner Y, Bendikov M (2010) $\alpha$-Oligofurans. Journal of the American Chemical Society 132: 2148–2150.

6. Diaz A, Crowley J, Bargon J, Gardini G, Torrance J (1981) Electrooxidation of aromatic oligomers and conducting polymers. Journal of Electroanalytical Chemistry 121: 355–361.

7. Jasti R, Bhattacharjee J, Neaton JB, Bertozzi CR (2008) Synthesis, characterization, and theory of [9]-, [12]-, and [18]Cycloparaphenylene: carbon nanohoop structures. Journal of the American Chemical Society 130: 17646–17647.

8. Davis W, Svec W, Ratner M, Wasielewsk M (1998) Molecular-wire behaviour in p-phenylene-vinylene oligomers. Nature 396: 60.

9. Zotti G, Martina S, Wegner G, Schlter A (1992) Well-defined pyrrole oligomers: Electrochemical and UV/vis studies. Advanced Materials 4: 798–801.

10. Becker RS, de Melo JS, Macanita AL, Elisei F (1996) Comprehensive evaluation of the absorption, photophysical, energy transfer, structural, and theoretical properties of -Oligothiophenes with one to seven rings. The Journal of Physical Chemistry 100: 18683–18695.

11. van Pham C, Burkhardt A, Shabana R, Cunningham DD, Mark HB, et al. (1989) A convenient synthesis of 2,5-Thienylene oligomers; some of their spectroscopic and electrochemical properties. Phosphorus, Sulfur, and Silicon and the Related Elements 46: 153.

12. Grebner D, Helbig M, Rentsch S (1995) Size-Dependent properties of oligothiophenes by picosecond Time-Resolved spectroscopy. The Journal of Physical Chemistry 99: 16991–16998.

13. Bäuerle P, Fischer T, Bidlingmeier B, Rabe JP, Stabel A (1995) Oligothiophenes — Yet longer? Synthesis, characterization, and scanning tunneling microscopy images of homologous, isomerically pure oligo(alkylthiophene)s. Angewandte Chemie International Edition in English 34: 303–307.

14. Frère P, Raimundo J, Blanchard P, Delaunay J, Richomme P, et al. (2003) Effect of local molecular structure on the chain-length dependence of the electronic properties of thiophene-based $\pi$-conjugated systems. The Journal of Organic Chemistry 68: 7254–7265.

15. Walker IC, Palmer MH, Hopkirk A (1989) The electronic states of the azines. II. Pyridine, studied by VUV absorption, near-threshold electron energy loss spectroscopy and ab initio multi-reference configuration interaction calculations. Chemical Physics 141: 365–378.

16. Pan H, Li Y, Wu Y, Liu P, Ong BS, et al. (2006) Synthesis and thin-film transistor performance of poly(4,8-didodecylbenzo[1,2-b:4,5-b$'$]dithiophene). Chemistry of Materials 18: 3237–3241.

17. Hou J, Park M, Zhang S, Yao Y, Chen L, et al. (2008) Bandgap and molecular energy level control of conjugated polymer photovoltaic materials based on benzo[1,2-b:4,5-b′]dithiophene. Macromolecules 41: 6012-6018.

18. Amer A, Burkhardt A, Nkansah A, Shabana R, Galal A, et al. (1989) Studies of some hindered 2,2′-bithienyls and 3,3′-bridged 2,2′-bithienyls with special reference to their uv spectra and oxidation potentials. Phosphorus Sulfur Silicon Relat Elem 42: 63-71.

19. Roncali J, Thobie-Gautier C (1994) An efficient strategy towards small bandgap polymers: The rigidification of the -conjugated system. Advanced Materials 6: 846-848.

20. Nishide Y, Osuga H, Saito M, Aiba T, Inagaki Y, et al. (2007) Synthesis and properties of a series of Well-Defined and polydisperse benzo[1,2-b:4,3-b]dithiophene oligomers. The Journal of Organic Chemistry 72: 9141–9151.

21. Mitsudo K, Shimohara S, Mizoguchi J, Mandai H, Suga S (2012) Synthesis of nitrogen-bridged terthiophenes by tandem buchwald-hartwig coupling and their properties. Org Lett 11: 2702-2705.

22. Morin JF, Leclerc M (2002) 2,7-carbazole-based conjugated polymers for blue, green, and red light emission. Macromolecules 35: 8413-8417.

23. Wakim S, Blouin N, Gingras E, Tao Y, Leclerc M (2007) Poly(2,7-carbazole) derivatives as semiconductors for organic thin-film transistors. Macromol Rapid Commun 28: 1798-1803.

24. Zhang ZB, Fujiki M, Tang HZ, Motonaga M, Torimitsu K (2002) The first high molecular weight poly(n-alkyl-3,6-carbazole)s. Macromolecules 35: 1988-1990.

25. Beaupré S, Leclerc M (2003) Optical and electrical properties of -conjugated polymers based on electron-rich 3,6-dimethoxy-9,9-dihexylfluorene unit. Macromolecules 36: 8986-8991.

26. Pei J, Yu WL, Huang W, Heeger AJ (2000) The synthesis and characterization of an efficient green electroluminescent conjugated polymer: poly[2,7-bis(4-hexylthienyl)-9,9-dihexylfluorene]. Chem Commun 32: 1631-1632.

27. Zhang X, Matzger AJ (2003) Effect of ring fusion on the electronic absorption and emission properties of oligothiophenes. Journal of Organic Chemistry 68: 9813–9815.

28. Zhang X, Köhler M, Matzger AJ (2004) Alkyl-substituted thieno[3,2-b]thiophene polymers and their dimeric subunits. Macromolecules 37: 6306-6315.

29. Zhang X, Ct AP, Matzger AJ (2005) Synthesis and structure of fused -Oligothiophenes with up to seven rings. Journal of the American Chemical Society 127: 10502–10503.

30. Henssler JT, Zhang X, Matzger AJ (2009) Thiophene/thieno[3,2-b]thiophene co-oligomers: Fused-ring analogues of sexithiophene. J Org Chem 74: 9112-9119.

31. Li Y, Wu Y, Liu P, Birau M, Pan H, et al. (2006) Bandgap and molecular energy level control of conjugated polymer photovoltaic materials based on benzo[1,2-b:4,5-b′]dithiophene. Adv Mater 18: 3029-3032.

32. Pomerantz M, Chaloner-Gill B, Harding LO, Tseng JJ, Pomerantz WJ (1993) New processable low band-gap, conjugated polyheterocycles. Synthetic Metals 55: 960–965.

33. Guo X, Ortiz RP, Zheng Y, Kim MG, Zhang S, et al. (2011) Thieno[3,4-c]pyrrole-4,6-dione-based polymer semiconductors: Toward high-performance, air-stable organic thin-film transistors. Journal of American Chemical Society 133: 13685-13697.

34. Shimizu Y, Shen Z, Ito S, Uno H, Daub J, et al. (2002) A convenient synthesis of isothianaphthene oligomers and their electrochemical studies. Tetrahedron Letters 43: 8485–8488.

35. Quattrocchi C, Lazzaroni R, Bredas JL, Kiebooms R, Vanderzande D, et al. (1995) Optical absorption spectra of aromatic isothianaphthene oligomers: Theory and experiment. Journal of Physical Chemistry 99: 3932–3938.

36. Nishiyama K, Honda T, Reis H, Mller U, Mllen K, et al. (1998) Electronic structures of 9,10-Anthrylene dimers and trimers in solution: formation of charge separation states depending on alkyl substituent groups. Journal of Physical Chemistry A 102: 2934–2943.

37. Weitzel H, Bohen A, Mllen K (1990) Polyarylenes and poly(arylenevinylene)s, 3. oligomeric model compounds for poly(9,10anthrylenevinylene). Die Makromolekulare Chemie 191: 2815–2835.

38. Bae WJ, Scilla C, Duzhko VV, Ho Jo W, Coughlin EB (2011) Synthesis and photophysical properties of soluble lowbandgap thienothiophene polymers with various alkyl sidechain lengths. Journal of Polymer Science Part A: Polymer Chemistry 49: 3260–3271.

39. Büschel M, Stadler C, Lambert C, Beck M, Daub J (2000) Heterocyclic quinones as core units for redox switches: UVvis/NIR, FTIR spectroelectrochemistry and DFT calculations on the vibrational and electronic structure of the radical anions. Journal of Electroanalytical Chemistry 484: 24–32.

40. Becker RS, Singh IS, Jackson EA (1963) Comprehensive spectroscopic investigation of polynuclear aromatic hydrocarbons. I. Absorption spectra and state assignments for the tetracyclic hydrocarbons and their alkylsubstituted derivatives. The Journal of Chemical Physics 38: 2144–2171.

41. Karcher W, Fordham R, Dubois J, Claude P, Ligthart J, editors (1985) Spectral atlas of polycyclic aromatic compounds: including data on occurrence and biological activity, volume 3 of *Spectral Atlas of Polycyclic Aromatic Compounds Series*. Dordrecht, Netherlands: D. Reidel.

42. Jellison JL, Lee CH, Zhu X, Wood JD, Plunkett KN (2012) Electron acceptors based on an all-carbon donoracceptor copolymer. Angewandte Chemie International Edition 51: 12321-12324.

43. Andrews L, Kelsall B, Blankenship T (1982) Vibronic absorption spectra of naphthalene and substituted naphthalene cations in solid argon. The Journal of Physical Chemistry 86: 2916–2926.

44. Chandross EA, Ferguson J, McRae EG (1966) Absorption and emission spectra of anthracene dimers. The Journal of Chemical Physics 45: 3546.

45. Rang Z, Haraldsson A, Kim D, Ruden P, Nathan M, et al. (2001) Hydrostatic-pressure dependence of the photoconductivity of single-crystal pentacene and tetracene. Applied Physics Letters 79: 2731.

46. Hinderhofer A, Heinemeyer U, Gerlach A, Kowarik S, Jacobs R, et al. (2007) Optical properties of pentacene and perfluoropentacene thin films. The Journal of Chemical Physics 127: 194705.

47. Payne M, Parkin S, Anthony J (2005) Functionalized higher acenes: Hexacene and heptacene. Journal of the American Chemical Society 127: 8028–8029.

48. Khan ZH, Khanna BN (1973) Electronic absorption spectra of pyrene and its monopositive ion. The Journal of Chemical Physics 59: 3015–3019.

49. van Mullekom HAM, Vekemans JAJM, Meijer EW (1998) Bandgap engineering of donoracceptor-substituted $\pi$conjugated polymers. Chemistry - A European Journal 4: 1235–1243.

50. Karikomi M, Kitamura C, Tanaka S, Yamashita Y (1995) New Narrow-Bandgap polymer composed of benzobis(1,2,5-thiadiazole) and thiophenes. Journal of the American Chemical Society 117: 6791–6792.

51. Kitamura C, Tanaka S, Yamashita Y (1996) Design of narrow-bandgap polymers. syntheses and properties of monomers and polymers containing aromatic-donor and o-quinoid-acceptor units. Chem Mater 8: 570-578.

52. Tanaka S, Yamashita Y (1995) Syntheses of narrow band gap heterocyclic copolymers of aromatic-donor and quinonoid-acceptor units. Synthetic Metals 69: 599-600.

53. Tanaka S, Yamashita Y (1993) Synthesis of a narrow band gap heterocyclic polymer: Poly-4,6-di(2-thienyl)thieno[3,4-c][1,2,5]thiadiazole. Synthetic Metals 55: 1251–1254.

54. Berton N, Ottone C, Labet V, de Bettignies R, Bailly S, et al. (2011) New alternating copolymers of 3,6-carbazoles and dithienylbenzothiadiazoles: Synthesis, characterization, and application in photovoltaics. Macromol Chem Phys 212: 2127-2141.

55. Wang E, Ma Z, Zhang Z, Vandewal K, Henriksson P, et al. (2011) An easily accessible isoindigo-based polymer for high-performance polymer solar cells. J Am Chem Soc 133: 14244-14247.

56. Nie WY, MacNeill CM, Li Y, Noftle RE, Carroll DL, et al. (2011) A soluble high molecular weight copolymer of benzo[1,2-b:4,5-b$'$]dithiophene and benzoxadiazole for efficient organic photovoltaics. Macromol Rapid Commun 32: 1163-1168.

57. Hellström S, Zhang FL, Inganäs O, Andersson MR (2009) Structure-property relationships of small bandgap conjugated polymers for solar cells. Dalton Trans 2009: 10032-10039.

58. Price SC, Stuart AC, You W (2010) Low band gap polymers based on benzo[1,2-b:4,5-b′]]di-thiophene: Rational design of polymers leads to high photovoltaic performance. Macromolecules 43: 4609-4612.

59. Price SC, Stuart AC, You W (2011) Development of fluorinated benzothiadiazole as a structural unit for a polymer solar cell of 7 %efficiency. Angew Chem Int Ed 50: 2995-2998.

60. Li Z, Zhang Y, Tsang SW, Du X, Zhou J, et al. (2011) Alkyl side chain impact on the charge transport and photovoltaic properties of benzodithiophene and diketopyrrolopyrrole-based copolymers. J Phys Chem C 115: 18002-18009.

61. Zhou HX, Yang L, Price SC, Knight KJ, You W (2010) Enhanced photovoltaic performance of low-bandgap polymers with deep lumo levels. Angew Chem Int Ed 49: 7992-7995.

62. Douglas JD, Griffini G, Holcombe TW, Young EP, Lee OP, et al. (2012) Functionalized isothianaphthene monomers that promote quinoidal character in donor–acceptor copolymers for organic photovoltaics. Macromolecules 2012 45: 4069-4074.

63. Lindgren LJ, Zhang FL, Andersson M, Barrau S, Hellström S, et al. (2009) Synthesis, characterization, and devices of a series of alternating copolymers for solar cells. Chem Mater 21: 3491-3502.

64. Piliego C, Holcombe TW, Douglas JD, Woo CH, Beaujuge PM, et al. (2010) Synthetic control of structural order in n-alkylthieno[3,4-c]pyrrole-4,6-dione-based polymers for efficient solar cells. J Am Chem Soc 132: 7595-7597.

65. Hellström S, Lindgren LJ, Zhou Y, Zhang FL, Inganäs O, et al. (2010) Side-chain architectures of 2,7-carbazole and quinoxaline-based polymers for efficient polymer solar cells. Polym Chem 39: 1272-1280.

66. Hwang Y, Kim FS, Xin H, Jenekhe SA (2012) New thienothiadiazole-based conjugated copolymers for electronics and optoelectronics. Macromolecules 45: 3732-3739.

67. Blouin N, Michaud A, Gendron D, Wakim S, Blair E, et al. (2008) Toward a rational design of poly(2,7-carbazole) derivatives for solar cells. J AM CHEM SOC 130: 732-742.

68. Wang EG, Hou LT, Wang ZQ, Ma ZF, Hellström S, et al. (2011) Side-chain architectures of 2,7-carbazole and quinoxaline-based polymers for efficient polymer solar cells. Macromolecules 44: 2067-2073.

69. Zhang Y, Zou J, Yip HL, Sun Y, Davies JA, et al. (2011) Conjugated polymers based on c, si and n-bridged dithiophene and thienopyrroledione units: synthesis, field-effect transistors and bulk heterojunction polymer solar cells. J Mater Chem 21: 3895-3902.

70. Bijleveld JC, Zoombelt AP, Mathijssen SGJ, Wienk MM, Turbiez M, et al. (2009) Poly(diketopyrrolopyrrole-terthiophene) for ambipolar logic and photovoltaics. J Am Chem SOC 131: 16616-16617.

71. Bijleveld JC, Gevaerts VS, Nuzzo DD, Turbiez M, Mathijssen SGJ, et al. (2010) Efficient solar cells based on an easily accessible diketopyrrolopyrrole polymer. Adv Mater 22: 242-246.

72. Cai TQ, Zhou Y, Wang EG, Hellström S, Zhang FL, et al. (2010) Low bandgap polymers synthesized by fecl3 oxidative polymerization. Solar Energy Materials and Solar Cells 94: 1275-1281.

73. Kawatsuki N, Hasegawa T, Ono H, Tamoto T (2003) Formation of polarization gratings and surface relief gratings in photocrosslinkable polymer liquid crystals by polarization holography. Adv Mater 15: 991-994.

74. Campbell AJ, Bradle DDC, Antoniadis H (2001) Dispersive electron transport in an electroluminescent polyfluorene copolymer measured by the current integration time-of-flight method. Appl Phys Lett 79: 2133-2135.

75. Stevens MA, Silva C, Russell DM, Friend RH (2001) Exciton dissociation mechanisms in the polymeric semiconductors poly9,9-dioctylfluorene... and poly9,9-dioctylfluorene-co-benzothiadiazole. PHYSICAL REVIEW B 63: 165213.

76. Kmínek I, Výprachtický D, Jaroslavkříž, Dybal J, Cimrová V (2010) Low-band gap copolymers containing thienothiadiazole units: Synthesis, optical, and electrochemical properties. Journal of Polymer Science Part A: Polymer Chemistry 48: 2743-2756.

77. Cimrová V, Kmínek I, Pavlačková P, Výprachtichý D (2011) Low-bandgap donoracceptor copolymers with 4,6-bis(3′-(2-ethylhexyl)thien-2′-yl)thieno[3,4-c][1,2,5]thiadiazole: Synthesis, optical, electrochemical, and photovoltaic properties. Journal of Polymer Science Part A: Polymer Chemistry 49: 3426-3436.

78. Lu JP, Liang FS, Drolet N, Ding JF, Tao Y, et al. (2008) Crystalline low band-gap alternating indolocarbazole and benzothiadiazole-cored oligothiophene copolymer for organic solar cell applications. Chem Commun 2008: 5315-5317.

79. Liang Y, Wu Y, Feng D, Tsai ST, Son HJ, et al. (2009) Development of new semiconducting polymers for high performance solar cells. J AM CHEM SOC 131: 56-57.

80. Bronstein J, Chen Z, Ashraf RS, Zhang W, Du J, et al. (2011) Thieno[3,2-b]thiophene-diketopyrrolopyrrole-containing polymers for high-performance organic field-effect transistors and organic photovoltaic devices. J Am Chem Soc 133: 3272-3275.

81. Wang EG, Hou L, Wang ZG, Hellström S, Mammo W, et al. (2010) Small band gap polymers synthesized via a modified nitration of 4,7-dibromo-2,1,3-benzothiadiazole. Org Lett 12: 4470-4473.

82. Wang E, Hou LT, Wang ZQ, Hellstrm S, Zhang F, et al. (2010) An easily synthesized blue polymer for high-performance polymer solar cells. Adv Mater 2010 22: 5240-5244.

83. Keshavarz-K M, Knight B, Haddon RC, Wudl F (1996) Syntheses of narrow band gap heterocyclic copolymers of aromatic-donor and quinonoid-acceptor units. Tetrahedron 52: 5149-5159.

84. Yang Y, Arias F, Echegoyen L, Chibante LPF, Flanagan S, et al. (1995) Reversible fullerene electrochemistry: Correlation with the homo-lumo energy difference for $c_{60}$, $c_{76}$,$cc_{78}$,$7c_{84}$. J Am Chem SOC 117: 7801-7804.

85. Sariciftci NS, Smilowitz L, Heeger AJ, Wudl F (1992) Photoinduced electron transfer from a conducting polymer to buckminsterfullerene. Science 258: 1474-1476.

86. Shin Y, Lin X (2013) Modeling photoinduced charge transfer across -conjugated heterojunctions. The Journal of Physical Chemistry C 117: 12432–12437.
